# Electrospun Nanofibers Loaded with *Verbena officinalis* Extract as Multifunctional Bioactive Wound Dressings

**DOI:** 10.3390/ph19081278

**Published:** 2026-08-13

**Authors:** Nikoleta Đorđevski, Ana Ćirić, Uroš Gašić, Marija Ivanov, Alexia Delnatte, Mikhael Bechelany, Dina Tucović, Jelena Kulaš, Maja Čakić-Milošević, Dejan Stojković

**Affiliations:** 1 Laboratory of Immunology, Institute of Microbiology, Medical Military Academy, 11000 Belgrade, Serbia; nikoleta.djordjevski@gmail.com; 2 Institute for Biological Research “Siniša Stanković”/National Institute of the Republic of Serbia, University of Belgrade, Bulevar Despota Stefana 142, 11108 Belgrade, Serbia; rancic@ibiss.bg.ac.rs (A.Ć.); uros.gasic@ibiss.bg.ac.rs (U.G.); marija.smiljkovic@ibiss.bg.ac.rs (M.I.);; 3 Institut Européen des Membranes, IEM, UMR 5635, Université Montpellier, CNRS, ENSCM, Place Eugène Bataillon, 34095 Montpellier, France; 4 Functional Materials Group, Gulf University for Science and Technology (GUST), Mubarak Al-Abdullah, Hawally 32093, Kuwait; 5 Institute of Zoology, Faculty of Biology, University of Belgrade, 11000 Belgrade, Serbia; 6 Department of Pharmacology, First Moscow State Medical University I.M. Sechenov (Sechenov University), Trubetskaya Street, House 8, Building 2, 119991 Moscow, Russia

**Keywords:** *Verbena officinalis*, skin health, antimicrobial, antibiofilm, nanofiber-based delivery, wound healing

## Abstract

**Background/Objectives**: *Verbena officinalis* has long been used in traditional medicine for treating skin disorders, yet its potential in advanced wound-dressing systems remains insufficiently explored. This study aimed to characterize *V. officinalis* extracts obtained using different solvents, evaluate their antimicrobial, antibiofilm, cytotoxic, and wound-healing activities, and develop electrospun polycaprolactone–polyethylene glycol (PCL–PEG) nanofibers as a delivery platform for the most active extract. **Methods**: Extracts were chemically characterized by LC–MS and evaluated against clinical bacterial, yeast, and dermatophyte isolates associated with skin infections. Antibiofilm activity was assessed against *Staphylococcus lugdunensis*. The most active hydroalcoholic extract (EW7.5) was incorporated into PCL–PEG nanofibers (1–10%, *w*/*w*). Metabolite release was monitored by semi-quantitative LC–MS analysis. Cytotoxicity and wound-healing activity were evaluated using HaCaT keratinocytes, while in vivo efficacy was assessed in a full-thickness excisional wound model in Dark Agouti rats (five animals per group in two independent experiments). **Results**: EW7.5 exhibited the strongest antimicrobial activity, with MIC values of 0.06 mg/mL against all tested bacterial and *Candida* strains and 0.06–0.5 mg/mL against dermatophytes. It inhibited *S. lugdunensis* biofilm formation by 79.43% at MIC/4 and reduced pre-formed biofilm biomass by 64.98% at 2 × MBC. EW7.5 and EW7.5-loaded nanofibers showed no cytotoxicity up to 400 μg/mL. In the scratch assay, the free extract achieved 42.34 ± 1.58% wound closure, while nanofibers containing 10% EW7.5 achieved 25.87 ± 2.52%, compared to 7.73 ± 0.07% for the control. In vivo, EW7.5-loaded membranes significantly accelerated wound closure and the 10% formulation significantly improved extracellular matrix deposition and vascular remodeling compared to the control membrane. **Conclusions**: EW7.5-loaded PCL–PEG nanofibers combine antimicrobial, antibiofilm, biocompatible, and wound-healing properties, supporting their potential as multifunctional wound dressings. Nevertheless, further studies with mechanistic investigations are required to fully establish their therapeutic applicability.

## 1. Introduction

Given the increasing resistance of microorganisms to existing drugs, alongside the rising incidence of adverse effects and the diminishing effectiveness of current treatments, the discovery of new pharmaceuticals represents one of the most significant challenges and pressing needs of the 21st century. There has been an increasing focus on natural products over the past decade in the pursuit of new medicinal therapies, particularly when combined with modern technology [1,2]. It is possible that the solution to this escalating issue may lie within folk medicine, where plants play a significant role since in some nations traditional medicine has established itself as a recognized form of treatment in regular medicine [3,4].

Ethnomedical records from the Balkan date back to the Middle Ages, with the Hilandar Medical Codex being a capital deed from that period. In this book, there are records of common vervain as a plant that was used to treat various skin damages and injuries [5]. Today, this plant is regarded as a “sacred herb” in European traditional medicine, according to well-documented ethnopharmacological records of its historical applications [6]. The Chinese Pharmacopeia and the European Pharmacopeia both list *Verbenae herba*, a raw material made up of the aerial parts [7]. Vervain, *Verbena officinalis* L. is part of the order Lamiales and is a cosmopolitan species with a wide distribution. *V. officinalis* is utilized in the treatment of various conditions, including diseases of the upper respiratory tract, particularly throat and sinus inflammation [8,9]. It is also employed for urinary tract disorders and menstrual irregularities, as well as to promote lactation during breastfeeding [10]. *V. officinalis* exhibits significant pharmacological effects, including antibacterial, antifungal, antioxidant, gastroprotective, and antiproliferative properties [11,12]. For skin conditions, it demonstrates anti-inflammatory and antibacterial effects, making it useful for treating difficult-to-heal wounds and gum inflammation. Topical applications of *V. officinalis* extracts can be effective for wounds, bites, oral cavity and throat inflammation, as well as muscle spasms [13]. Following oral administration, this extract reduces lung injury, promotes the maturation and activation of NK cells in the lungs, and decreases the levels of inflammatory cytokines (IL-6, TNF-α, IL-1β) in the serum [14]. Also, this extract alleviates neuro-inflammation following ischemic stroke by suppression of the IL-17A pathway [15]. *V. officinalis* contains a diverse array of bioactive components, which is related to its long-standing use in traditional medicine. Notable among these components are ursolic acid [16], phenylethanoid glycosides [17], iridoids [18], and flavonoids [11].

A variety of treatments are available to manage wounds and prevent infection. For over 2000 years, silver has been one of the most commonly used agents in wound care and continues to play a significant role in the treatment of wounds and skin injuries today [19]. Other methods include iodine-containing compounds [20], various topical antibiotics applied directly to the wound site [21], and moist wound dressings that help prevent infection and promote healing [22]. Traditional dry gauze, low-adherent dressings and semipermeable films are also widely used, along with hydrocolloids and hydrogels [23]. Several innovative treatments emphasize the integration of antimicrobial compounds directly into wound-dressing materials. These advanced dressings combine traditional wound care methods with foams and hydrogels infused with antimicrobial substances such as silver, betaine, chitin and others [23]. However, despite abundant therapeutics available for the treatment of skin infections, some cases like complicated skin and soft-tissue infections are more challenging to manage and highlight the need for the development of new antibiotics [24].

In recent years, electrospinning has emerged as a promising technique for fabricating nanofibrous materials that can serve as efficient drug delivery systems. This method enables the encapsulation of bioactive compounds within nanofibers composed of biodegradable and biocompatible polymers, allowing for controlled release and the localized application of therapeutic agents. Electrospun nanofibers offer a high surface-area-to-volume ratio, interconnected porosity and structural resemblance to the extracellular matrix, making them highly suitable for biomedical applications such as wound dressings, tissue engineering scaffolds and site-specific drug delivery. In addition, this technique helps protect sensitive natural compounds from environmental degradation while promoting sustained release at the wound site [25]. Polymers like polycaprolactone (PCL) and polyethylene glycol (PEG) are commonly used due to their mechanical strength, biocompatibility and ability to incorporate therapeutic agents [26].

This study aimed to evaluate the efficiency of different solvents in extracting biologically active compounds from *Verbena officinalis*, a medicinal plant traditionally used for the treatment of skin disorders according to the Hilandar medical codex. The objectives were to characterize the chemical profiles of the obtained extracts and to assess their biological activities, including antibacterial and antibiofilm effects against clinical bacterial and fungal isolates associated with superficial skin and soft-tissue infections. In addition, the cytotoxicity of both the extracts and the extract-loaded nanofiber membranes was evaluated on HaCaT keratinocyte cells, together with their in vitro wound-healing potential using a scratch assay model. Furthermore, the most active extract was incorporated into PCL–PEG electrospun nanofibers to develop a potential wound-healing material with antimicrobial properties, and its wound-healing efficacy was further investigated in vivo using a DA rat excisional wound model. To the best of our knowledge, this is the first study to integrate the comparative solvent-based phytochemical profiling of *V. officinalis*, biological selection of the most active extract against clinically relevant skin pathogens, fabrication of electrospun PCL–PEG nanofibers as a delivery platform, and validation of their wound-healing efficacy in both in vitro and in vivo models. Rather than focusing on a single aspect, this work establishes a translational workflow linking extract selection, chemical composition, antimicrobial efficacy, biomaterial development, and biological validation for topical wound-healing applications.

## 2. Results and Discussion

### 2.1. Chemical Analysis of the Extracts

The relative abundance of the identified metabolites was assessed semi-quantitatively using normalized LC-MS peak areas, allowing comparison of the major constituents among the different extracts. Chemical analysis of the *V. officinalis* extracts led to the identification of different classes of chemical constituents such as benzoic acid derivatives, cinnamic acid derivatives, iridoid glycosides, phenylethanoids, flavonoid *C*-glycosides, flavonoid *O*-glycosides, flavonoid aglycones, and xanthones (Table 1). The relative distribution of the main groups of compounds among the extracts was assessed semi-quantitatively based on LC–MS peak areas and visualized using heatmaps (Figures S1–S8). When it comes to benzoic acid derivatives, the most abundant representatives were dihydroxybenzoic acid pentosyl-pentoside found in the extracts obtained with ethanol–water mixtures (EW7.5, EW5, and EW2.5, Figure S1). Likewise, these extracts were rich in hydroxybenzoic acid isomer 2. In the case of cinnamic acid derivatives, the presence of caffeic acid, *p*-coumaric, and ferulic acids was detected in all the examined extracts, while extract EW2.5 was especially rich in ethyl caffeate (Figure S2). Among the iridoid glycosides, we could note the abundant presence of verbenalin + HCOOH especially in water–ethanol extracts (EW7.5, EW5, and EW2.5) (Figure S3). Moreover, all extracts were rich in hastatoside + HCOOH and slightly less in brasoside + HCOOH (Figure S4). On the other hand, extract EW7.5 and EW5 were rich in verbascoside. Moreover, in these extracts along with EW2.5, we could detect significant levels of martynoside (Figure S4). Among the flavonoid *C*-glycosides we could observe high levels of apigenin 6,8-di-*C*-hexoside, especially in EW7.5, EW5, and EW2.5 (Figure S5). Chrysin 6,8-di-*C*-hexoside were on the other hand highest in the EW5 extract. In the case of flavonoid *O*-glycosides extracts, EW7.5, EW5, and EW2.5 were the most abundant in luteolin 7-*O*-(2”-hexuronyl)-hexuronide, luteolin 7-*O*-hexuronide, and apigenin 7-*O*-hexuronide (Figure S6). On the other hand, extract W1 had the lowest levels of detectable flavonoid *O*-glycosides. Among the aglycones of flavonoids detected in the extracts, the levels of luteolin were the highest, especially in EW2.5 (Figure S7). Flavonoid aglycones present in all the extracts at lower levels were isorhamnetin, hispidulin, tricin, cirsimaritin, and genkwanin. The lowest diversity of the flavonoid aglycones could be noted in W1. Xanthones that were detected in all the extracts were trihydroxy-methoxyxanthone, dihydroxy-dimethoxyxanthone, trihydroxy-dimethoxyxanthone, and trihydroxy-trimethoxyxanthone (Figure S8). These results demonstrate a clear solvent-dependent distribution of chemical classes, with ethanol–water mixtures favoring the extraction of iridoid glycosides, phenylpropanoids, and flavonoids, while the aqueous extract showed the lowest chemical diversity and relative abundance. Based on this semi-quantitative analysis, EW7.5 exhibited the highest relative abundance of verbenalin, hastatoside, verbascoside, flavonoid glycosides, and some phenolic acid derivatives, which contributed to its selection for further biological evaluation.

Different extracts of *V. officinalis* have been previously studied, and their chemical composition was revealed. In one of the chemical evaluations, petroleum ether and chloroform extracts were used for the isolation of diverse compounds such as β-sitosterol, ursolic acid, oleanolic acid, 3-epiursolic acid, 3-epioleanolic acid, while methanol extract served as the source of two iridoid glucosides, verbenalin and hastatoside, a phenylpropanoid glycoside, verbascoside and β-sitosterol-D-glucoside [27]. Similarly to the methanol extract in the previous study, extracts obtained with ethanol and water mixture were noted as the rich sources of verbenalin (Figure S3). The chemical composition of plant extract obtained with 70% ethanol was examined in Dai et al. [14]. The study has identified different molecules including 3,4-dihydroverbenalin, verbeofflin I, hastatoside, verbenalin, and other. Similarly, our study has obtained extract EW7.5 with 75% ethanol and it was also rich in hastatoside and verbenalin. The detailed study of *V. officinalis* aqueous and hydroalcoholic extracts was performed in Rehecho et al. [28], where the presence of three iridoids, fifteen flavonoids and four phenolic acid derivatives was noted. Moreover, four flavonoids, scutellarein 7-diglucuronide, scutellarein 7-glucuronide, pedalitin 6-galactoside and scutellarein 7-glucoside, were observed for the first time in *V. officinalis*. Some other constituents in the extract such as hastatoside, luteolin 7-*O*-glucoside and verbenalin [28] were present similarly to our study (Table 1).

It seems that the combination of ethanol and water leads to a higher yield of extracted constituents of *V. officinalis* (Figures S1–S8). This is in accordance with the previous study of Ivanov et al. [29] which highlighted ethanol–water solvents as the ones that tend to increase amounts of extracted polyphenolic compounds. Another study has examined the effect of different solvent rations on the extraction of flaxseeds and indicated that the mixture of ethanol and water in the proportion of 60:40 (*v*/*v*) is the most efficient in order to achieve a high content of phenolic compounds [30].

### 2.2. Antibacterial Potential of Extracts

The ability of *V. officinalis* extracts to reduce the growth of skin pathogenic bacteria was evaluated by microdilution assay (Table 2). For bacteria strains, we could note strongest inhibitory capacity for extracts prepared with the combination of ethanol and water (EW7.5, EW5, and EW2.5 with MIC range 0.06–1 mg/mL) followed by the ethanol extract (E1 MICs 0.25–0.5 mg/mL), while the lowest antimicrobial capacity was recorded for water extract (W1 MIC 1–2 mg/mL). Although EW7.5 exhibited a lower MIC against *S. epidermidis* than streptomycin under the present experimental conditions, direct comparisons between crude plant extracts and purified antimicrobial agents should be interpreted with caution because they differ substantially in chemical complexity, active compound concentration, and mechanisms of action.

Ethanolic extract of leaves of *V. officinalis* and its fractions were examined towards different microorganisms [31]. Ethanolic extract displayed MIC in the range 0.8–3.3 mg/mL. Its MIC was 1.3 mg/mL [31] towards *S. epidermidis*, which is higher than the MIC observed in this study (E1, 0.25 mg/mL), possibly due to different origins and the different chemical composition of the extract.

### 2.3. Extracts as Inhibitors of Skin-Isolated Fungi

Five extracts of *V. oficianalis* were analyzed in order to evaluate their potential to inhibit the growth of dermatomycetes (Table 3) and yeast (Table 2). The most promising inhibitory potential could be observed for the EW7.5 extract, with MICs ranging 0.06–0.5 mg/mL for all tested dermatomycetes. Lowest MIC values were also noted in the case of extract EW7.5 and were 0.06 mg/mL towards all yeast strains examined, while *C. albicans* (Y177) and *C. krusei* (Y454) were especially sensitive to E1 and EW2.5, respectively (MIC 0.06 mg/mL). These lowest MIC values were observed for the EW7.5, and in some cases E1 and EW2.5 were lower than the MIC values of the positive control, commercial antifungal agent, ketoconazole. Strains that were most sensitive to the treatment belong to *T. rubrum* (D460 and D1026), while the most resistant to extract antifungal activity was strain *T. mentagrophytes* (D448).

The impact of different *V. officinalis* extracts on the growth of clinical isolates of dermatomycetes has not been examined before. EW7.5 superior anti-dermatomycete potential might be related to its higher levels of bioactive constituents (Figures S1–S7), but further research is needed in order to examine the anti-dermatomycetic potential of EW7.5 constituents and provide more insight into this relation. Additionally, not many plant extracts have been studied so far as inhibitors of dermatomycetes. A previous study of *Allium scorodoprasum* flower extracts revealed their MIC values in the range 0.25–4 mg/mL [32], while the lowest MIC values in this study were 0.06 mg/mL. For several tested strains, EW7.5 exhibited MIC values comparable to or lower than those of ketoconazole under the experimental conditions employed. However, these observations should not be interpreted as evidence of superior antifungal efficacy because crude plant extracts contain multiple constituents that differ fundamentally from purified reference antifungal drugs in composition, the concentration of active compounds, and pharmacological properties.

Also, this study has observed the strong capacity of *V. officinalis* to reduce the growth of different *Candida* species. Extracts most active in the inhibition of *Candida* were the ones obtained with water–ethanol mixture (Table 2). These extracts were also rich in luteolin and its derivatives (Figure S7), with lutolin being the flavonoid with confirmed inhibitory potential on different *Candida* species [33]. Further studies could highlight the anti-*Candida* potential of luteolin derivatives present in the *V. officinalis* extract which may provide deeper insights on the relationship between its chemical composition and bioactivity.

### 2.4. Antibiofilm Capacity of V. officinalis

**Figure 1:**
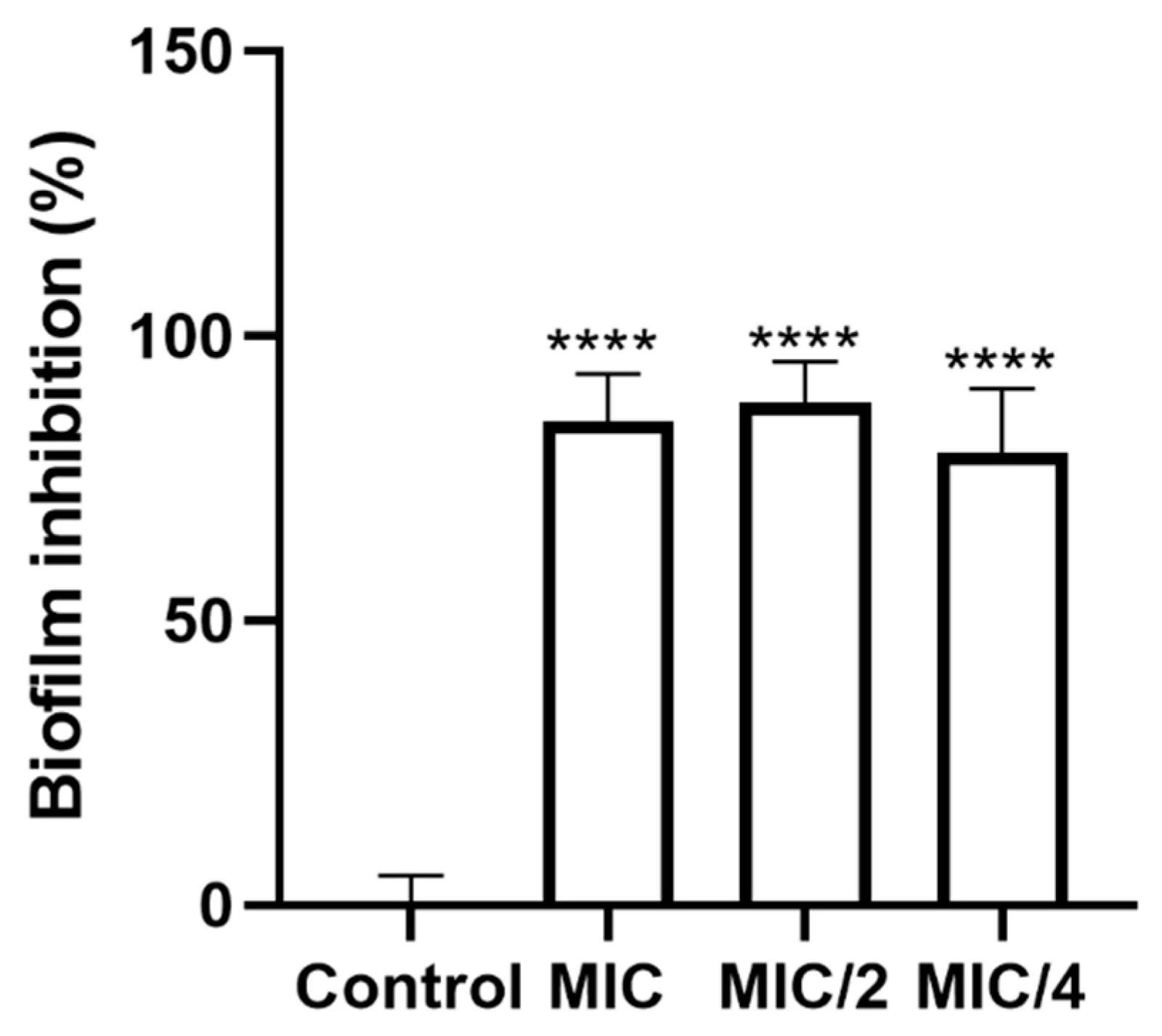
Inhibition of *S. lugdunensis* (B43) biofilm formation upon the addition of EW7.5 extract. The results are presented as the inhibition percentage of 3 replicates ± SD. The asterisks present statistical significance calculated in comparison of treatments with untreated control, **** *p* < 0.0001.

Due to its profound effect on the inhibition of microbial growth in the microdilution assay, extract EW7.5 was selected for the evaluation of antibiofilm potency. EW7.5 has shown significant antibiofilm potential towards *S. lugdunensis* (B43) (**** *p* < 0.0001, Figure 1). Even the lowest examined concentration (MIC/4) inhibited biofilm formation for 79.43%.

**Figure 2:**
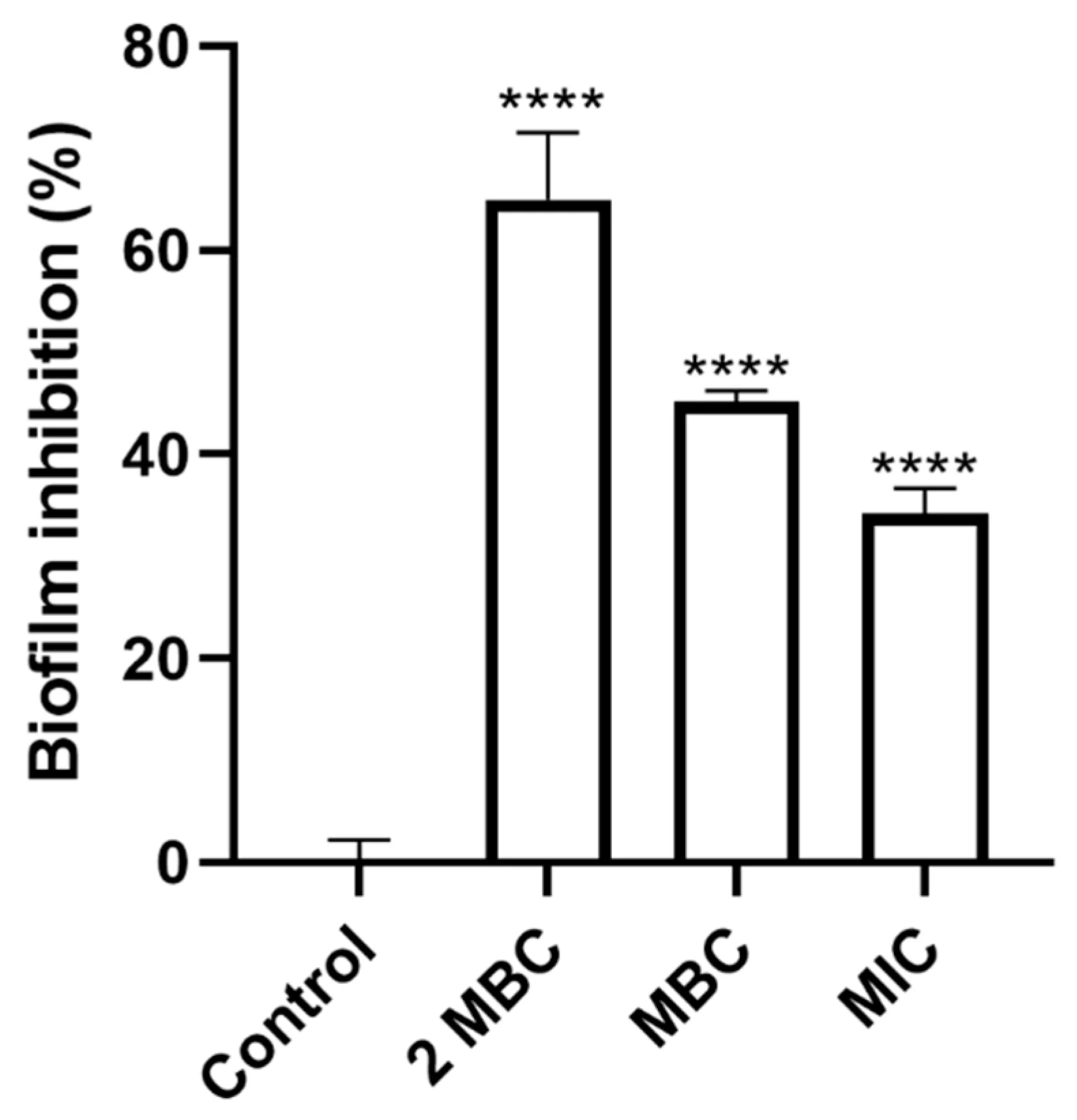
Inhibition of *S. lugdunensis* (B43) 24 h old biofilm biomass upon addition of the EW7.5 extract. The results are presented as the inhibition percentage of 3 replicates ± SD. The asterisks present statistical significance calculated in comparison of treatments with untreated control, **** *p* < 0.0001.

We have further evaluated the potential of EW7.5 to eradicate pre-formed biofilms of *S. lugdunensis* (B43) (Figure 2). The addition of EW7.5 to the 24 h old bacterial biofilm had led to the eradication of more than 50% of biofilm biomass (64.98% inhibition with 2 MBC). Moreover, the lowest concentration examined (MIC) had led to 34.17% inhibition.

The antibiofilm potential of *V. officinalis* examined in this study has revealed the great potential of this plant, more precisely the EW7.5 extract, to be included in some novel antibiofilm strategies since all the observed inhibitory activities were significant in comparison with the control (Figure 1 and Figure 2). The potential to inhibit bacterial biofilms was previously noted for some of the EW7.5 constituents such as luteolin [34], caffeic acid [35] and naringenin [36]. These encouraging results demonstrate the potential of *V. officinalis* EW7.5 extract as a possible element with a role in the creation of innovative antibiofilm strategies, necessitating additional research into its bioactive components and their modes of action.

### 2.5. Effect of EW7.5 Incorporation on the Fiber Physicochemical Properties

**Figure 3:**
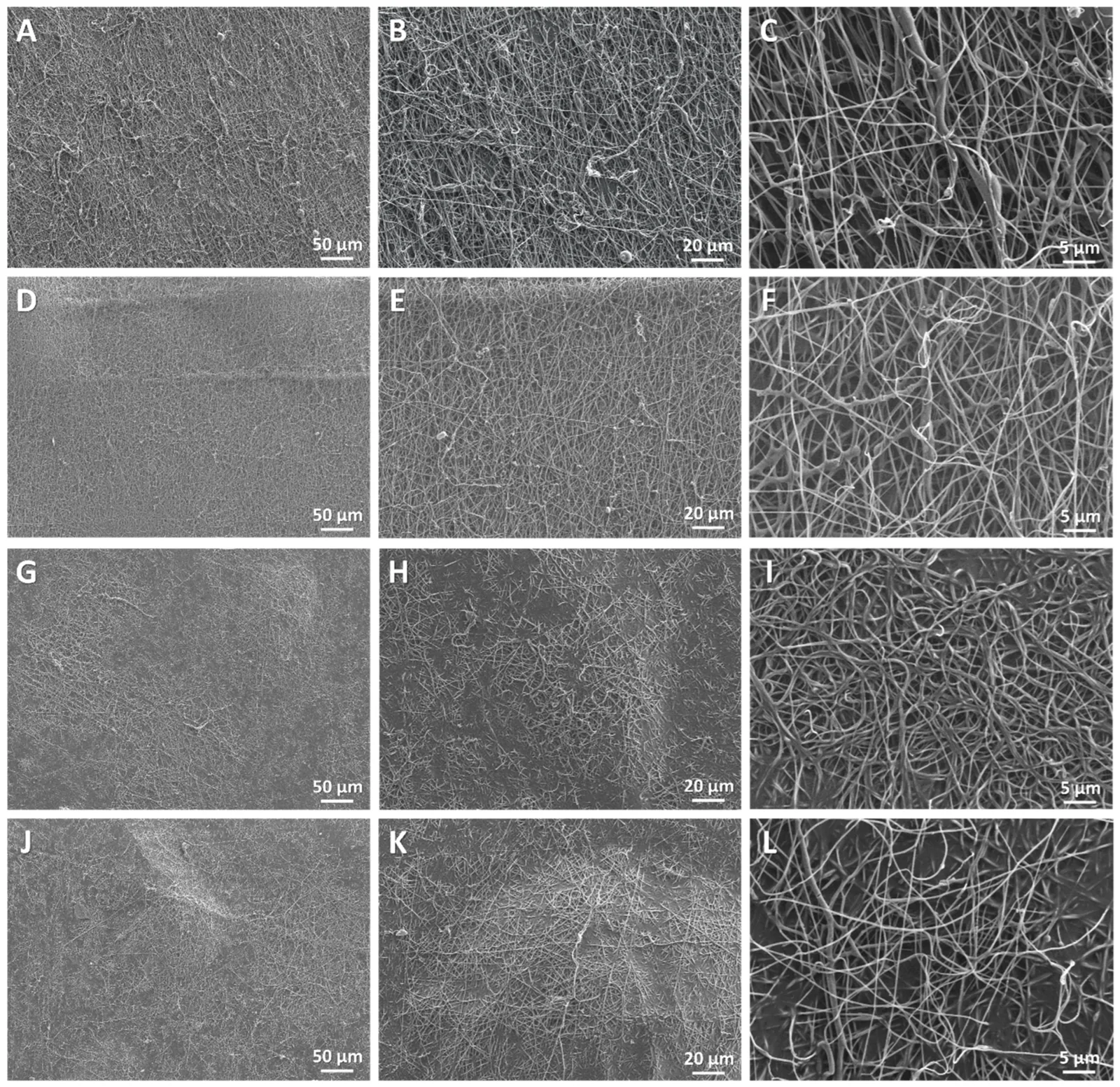
Characterization of electrospun nanofibers. SEM images of the membranes: (**A**–**C**) pure, (**D**–**F**) with 1% EW7.5, (**G**–**I**) with 5% EW7.5 and (**J**–**L**) with 10% EW7.5. Each set of panels shows images at scale bars of 50, 20 and 5 µm, respectively.

The morphology of the electrospun PCL-PEG nanofiber membranes without and with EW7.5 extract (1%, 5% and 10%) was observed using scanning electron microscopy (SEM), as shown in Figure 3A–L. The pure membrane (A) exhibits a continuous and interconnected network with a relatively heterogeneous distribution of fiber diameters (0.56 ± 0.34 µm). After the incorporation of the EW7.5 extract, the fibers (B–D) became thinner and more uniform, with average diameters of 0.42 ± 0.19 µm, 0.41 ± 0.21 µm and 0.40 ± 0.17 µm for 1%, 5% and 10% EW7.5, respectively.

The addition of the extract appeared to enhance fiber homogeneity and promote a slight reduction in fiber diameter, likely due to modifications in the electrospinning solution’s properties, such as viscosity and conductivity. As noted by Li et al. [37], the introduction of functional components into a polymeric solution can alter its physical characteristics, ultimately affecting the structure and morphology of the fibers. According to the previous study [38], the increase in conductivity due to the incorporation of additives, like polyethylene glycol (PEG) or dipyridamole (DPA), led to a reduction in fiber diameter, which is consistent with the results observed in this study. Specifically, the observed decrease in fiber diameter may be explained by an increase in the solution’s conductivity, which facilitates a greater transfer of electrical charges into the polymer jet. This enhanced conductivity promotes bending instability and further elongation of the jet during electrospinning, resulting in thinner fibers, as described by Lee et al. [39].

Interestingly, no significant structural defects or fiber agglomerations were observed across all samples, suggesting good compatibility between the polymer matrix and the EW7.5 extract.

Overall, SEM analysis indicates that EW7.5 incorporation improves the uniformity of the fibers while maintaining their morphological integrity across different concentrations. These observations provide a solid basis for further investigation of membrane chemical composition and mechanical properties.

**Figure 4:**
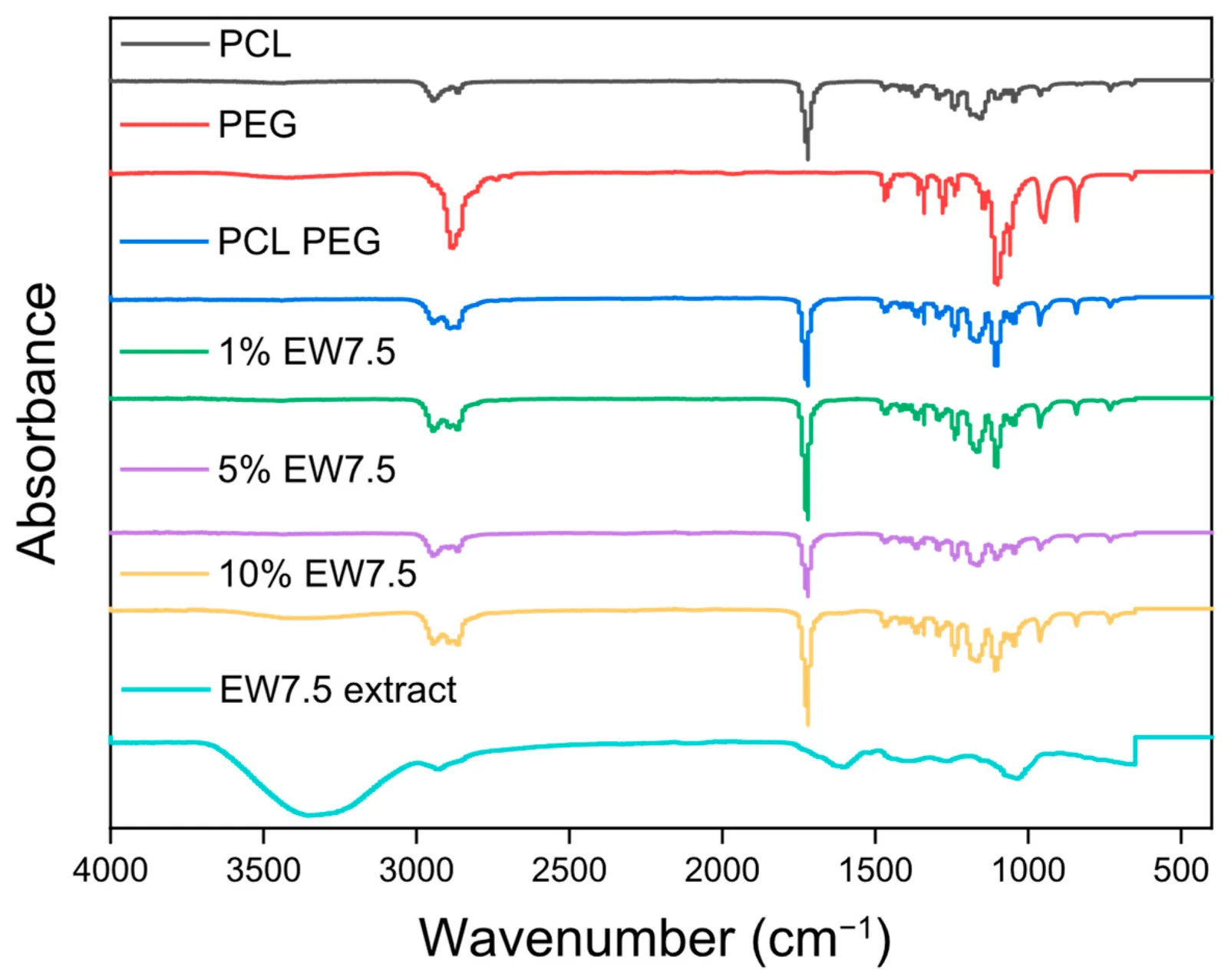
FTIR spectra of PCL, PEG, PCL/PEG blend, and nanofiber membranes loaded with EW7.5 at different concentrations (1%, 5% and 10%), as well as the pure extract.

FTIR was used to investigate the chemical structure of the electrospun membranes and to assess the presence of the EW7.5 extract within the nanofiber matrix (Figure 4). The spectra of pure PCL exhibit characteristic peaks at around 1720 cm^−1^ (C=O stretching of ester groups), 2940–2865 cm^−1^ (C−H stretching) and 1293–1160 cm^−1^ (C−O−C vibrations), which are typical of aliphatic polyesters. PEG displays a broad absorption band near 3400 cm^−1^ corresponding to O−H stretching and a strong band around 1100 cm^−1^ related to C−O−C ether linkages. The PCL/PEG blend shows the combined features of both polymers.

The FTIR spectrum of the pure EW7.5 extract reveals distinct bands at 3400 cm^−1^ (O−H stretching of phenolic groups), 1605 cm^−1^ and 1510 cm^−1^ (aromatic C=C stretching), and 1050–1250 cm^−1^ (C−O stretching), which are characteristic of polyphenolic compounds. In the extract-loaded nanofibers (EW7.5 1%, EW7.5 5% and EW7.5 10%), the main peaks of PCL and PEG remain visible, indicating that the polymer structure is preserved. Some of these extract-related peaks are partially retained. Notably, the O−H band around 3400 cm^−1^ is barely detectable at 1% and 5% concentrations but becomes more pronounced at 10%, suggesting that a minimum extract concentration is needed for this signal to be detectable. Furthermore, slight shifts and changes in the intensity of peaks in the 1600–1500 cm^−1^ region indicate possible interactions between the extract and the polymer matrix. These spectral changes confirm the successful incorporation of the EW7.5 extract into the nanofibers without significant alteration in the base polymer structure.

**Figure 5:**
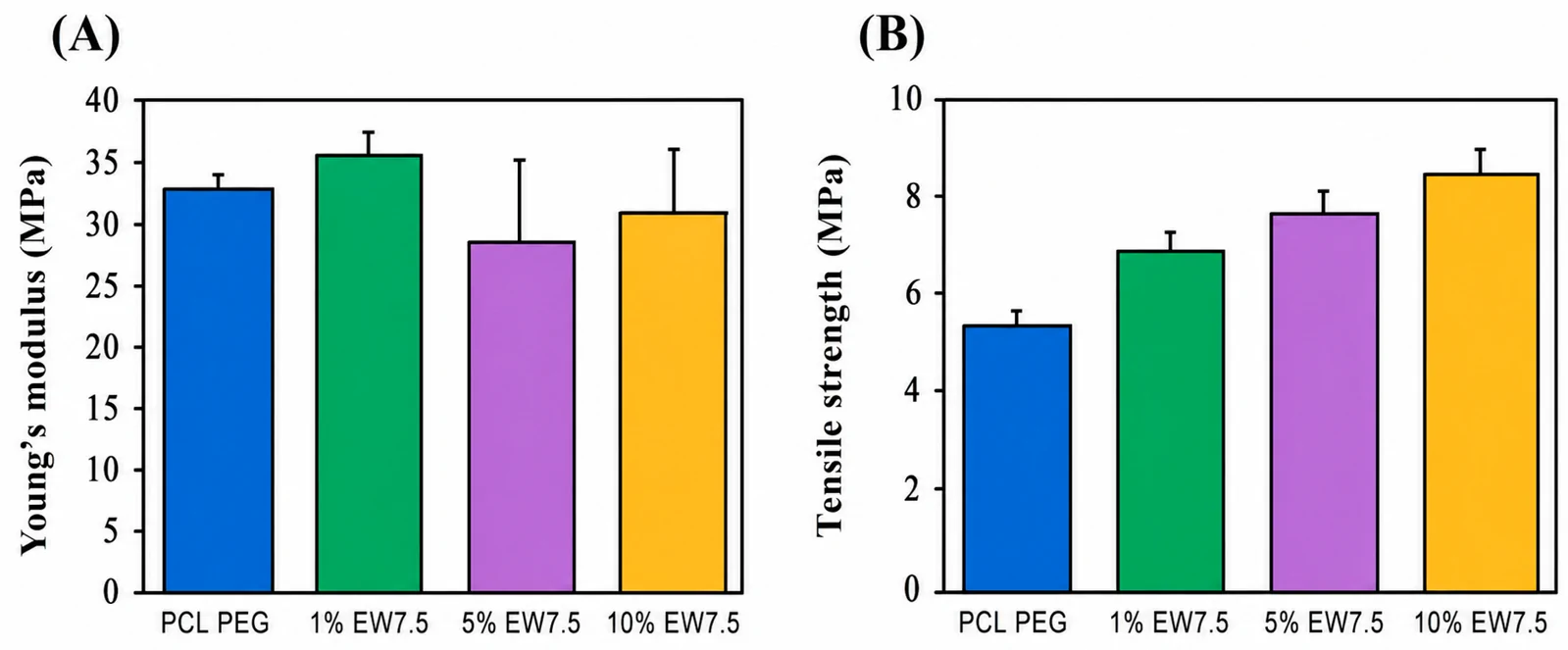
Mechanical properties of the membranes with different concentrations of EW7.5 extract. (**A**) Young’s modulus and (**B**) tensile strength of nanofibers without extract and with 1%, 5% and 10% extract-loaded fibers.

The mechanical properties of the PCL/PEG fibers were evaluated in terms of Young’s modulus and tensile strength to assess the effect of extract incorporation (Figure 5).

The Young’s modulus remained relatively stable across the different formulations. The control sample (PCL/PEG) had a modulus of 32.89 ± 0.85 MPa, which showed slight variations with the 1% extract (36.00 ± 2.56 MPa), 5% extract (29.03 ± 6.97 MPa) and 10% extract (31.02 ± 5.15 MPa). These fluctuations suggest that the extract does not significantly affect the stiffness of the fibers.

In contrast, the tensile strength increased with the extract content. The control sample exhibited a strength of 5.39 ± 0.24 MPa, which increased to 6.98 ± 0.52 MPa with 1% extract, 7.61 ± 0.67 MPa with 5% extract and 8.40 ± 0.66 MPa with 10% extract. This trend suggests that the extract reinforces the polymer matrix, likely by enhancing fiber cohesion and mechanical resistance. The results indicate a dose-dependent effect, with the highest concentration providing the best performance. These findings are consistent with previous studies. For example, in curcumin (Cur)-loaded PCL-PEG nanofibers, the incorporation of Cur led to increased tensile strength and elongation at break, possibly due to favorable interactions between Cur and the polymer chains [40]. Similarly, another study involving *Elaeagnus angustifolia* (EA)-loaded PCL-PEG-PCL (PCEC) nanofibers reported a significant improvement in tensile strength with increasing extract concentration, highlighting the reinforcing effect of bioactive compounds through interaction with the polymer network [41]. In addition to these effects, other factors may contribute to the observed mechanical improvement. As observed in the SEM analysis, the incorporation of the EW7.5 extract led to a slight reduction in fiber diameter, which can increase the surface area and promote greater chain entanglement, enhancing the tensile strength. Furthermore, better fiber alignment during electrospinning could increase the load-bearing capacity, further reinforcing the material. 

A total of eleven compounds were detected in the release media from membranes loaded with 1%, 5%, and 10% of the extract, as identified by LC–MS analysis (Table S1). Results are expressed as relative peak areas obtained by LC–MS analysis. The released compounds mainly belonged to iridoid glycosides and phenylethanoids, corresponding to the major constituents of the incorporated extract. The release profiles demonstrated a clear dependence on both membrane loading and incubation time. For all detected compounds, higher extract loading resulted in increased signal intensities in the release medium (10% > 5% > 1%). A gradual increase in released amounts was observed from 15 to 240 min, indicating the time-dependent diffusion of bioactive constituents from the membrane matrix. After 24 h, most compounds exhibited stabilization of the release profile, suggesting the establishment of an equilibrium state. Minor fluctuations observed at intermediate time points may be attributed to experimental variability and matrix effects associated with ionization during LC–MS detection. Overall, the results confirm the successful incorporation of the extract into the membranes and demonstrate a sustained and concentration-dependent release of the identified bioactive compounds.

### 2.6. Antimicrobial Potential of Membranes Incorporated with EW7.5

**Figure 6:**
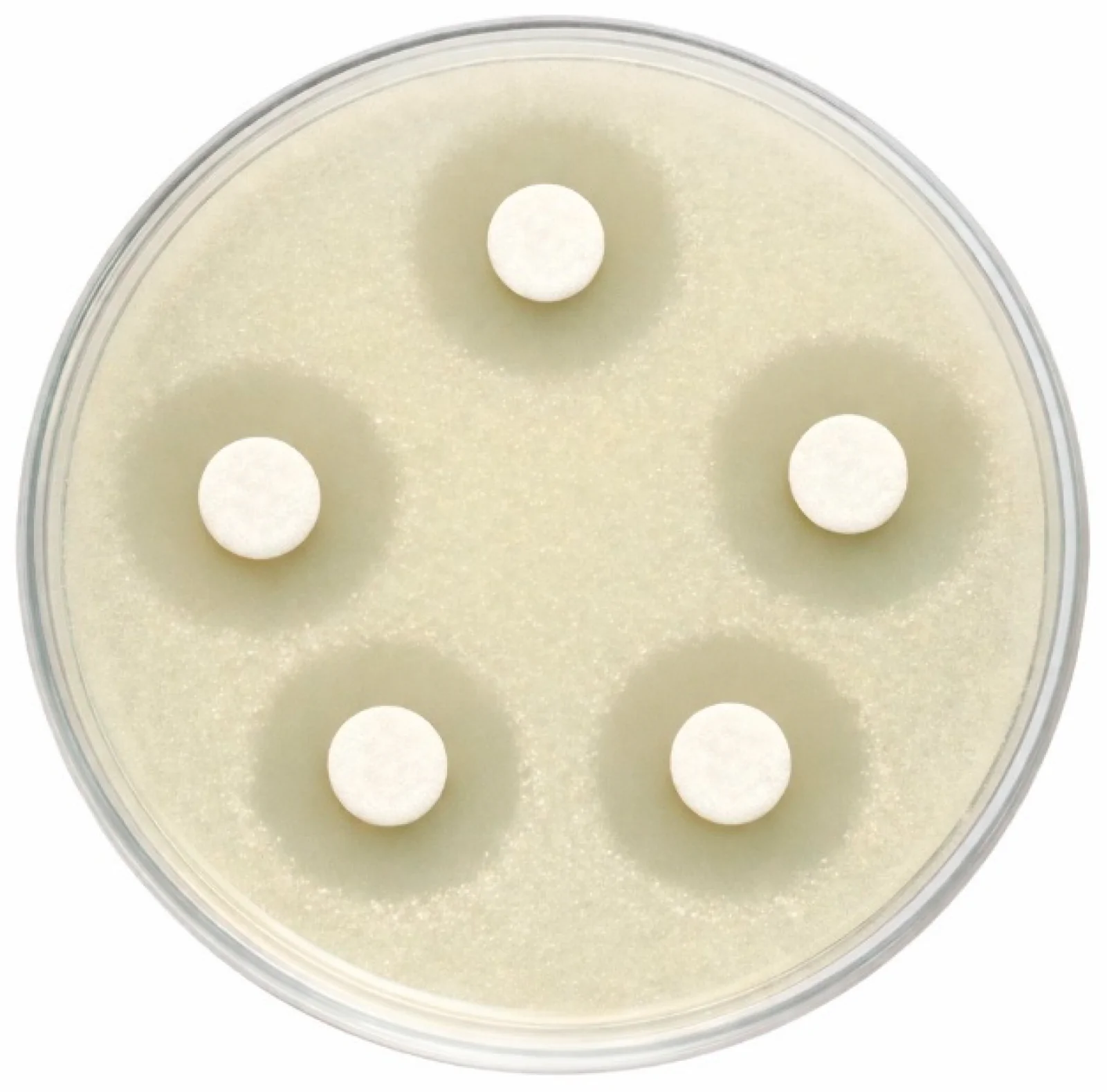
Representative photograph of inhibition zones obtained in the disk diffusion assay using electrospun PCL–PEG nanofibers incorporated with 5% EW7.5 against *Candida krusei* (Y454). All disks produced clearly defined and uniform inhibition zones with an average diameter of approximately 18 mm, indicating consistent antifungal activity of the EW7.5-loaded nanofibers.

Membranes incorporated with different concentrations of EW7.5 (1%, 5%, and 10%) have shown strong inhibitory potential towards different bacteria and yeasts (Table 4). In most of the cases, increased concentration of EW7.5 in the membranes can be linked to the wider inhibition zone. Inhibition zones greater than 30 mm were produced when membranes were incorporated with 10% EW7.5 towards *S. epidermidis* (B45) (31.62 mm), *P. vulgaris* (B44) (31.2 mm), and *C. kefyr* (Y289) (31.83 mm). Representative results of the disk diffusion assay are presented in Figure 6, demonstrating that PCL–PEG nanofibers containing 5% EW7.5 generate well-defined inhibition zones (~18 mm) against *Candida krusei*, in agreement with the antimicrobial activity values summarized in Table 4. The blank PCL–PEG membranes produced small inhibition zones in the assay. As no antimicrobial compounds were incorporated into these membranes, this effect is unlikely to reflect intrinsic antimicrobial activity of the polymer itself. Instead, it most likely results from physical or diffusion-related effects associated with the membrane matrix, as previously reported for polymeric materials evaluated using agar diffusion methods. The electrospun membranes were thoroughly dried prior to biological evaluation, making residual solvent an unlikely explanation for the observed inhibition zones. Statistical comparison with the blank PCL–PEG membranes confirmed a concentration-dependent contribution of EW7.5 to the observed antimicrobial activity. Membranes containing 10% EW7.5 produced significantly larger inhibition zones than blank membranes against all tested microorganisms (*p* < 0.001 or *p* < 0.0001), whereas the significance of the 1% and 5% formulations was microorganism-dependent.

This is the first study of the antimicrobial potential of membranes incorporated with *V. officinalis* extract with this novel delivery system being promising in the inhibition of microbial growth (Table 4). The review by Zhang et al. [42] provided information on the diverse antimicrobial plant extracts that when incorporated in electrospun nanofibers could be delivered in a controlled way. Potential applications of electrospun nanofibers with antimicrobial plant extracts could be in wound management, tissue engineering and the food industry [42]. Another review has highlighted the potential of electrospun nanofibers with bioactive natural products to prevent bacterial spreading at the initial site of infection in wounds and at the same time promote the healing of the skin [43]. Further studies of membranes with EW7.5 could involve in vivo experiments in order to confirm the efficacy of the novel designed membranes in the models of infected wounds.

### 2.7. Cytotoxicity of Extracts and Membranes

The cytotoxicity of the tested extracts (E1, EW7.5, EW5, EW2.5, and W1) and EW7.5-loaded electrospun membranes was assessed using the crystal violet assay on HaCaT keratinocyte cells. No cytotoxic effects were observed for any of the extracts at concentrations up to 400 µg/mL, and cell viability remained comparable to that of untreated control cells. Likewise, membrane leachates did not induce cytotoxicity, demonstrating good in vitro biocompatibility of the electrospun membranes under the experimental conditions. As no significant decrease in cell viability was detected for any sample or concentration, these results are described in the text and in the Table S2.

### 2.8. Wound-Healing Properties

The in vitro wound-healing activity of the tested extracts (E1, EW7.5, EW5, EW2.5, and W1) and EW7.5-loaded electrospun membranes on HaCaT keratinocyte cells after 24 h of treatment is presented in Table 5. All extracts stimulated keratinocyte migration to some extent; however, EW7.5 exhibited the strongest wound-healing effect, followed by EW5 and E1. In contrast, EW2.5 and W1 showed comparatively lower wound closure percentages. A similar trend was observed for membrane formulations, where increasing the amount of incorporated EW7.5 resulted in enhanced wound closure. Membranes containing 10% EW7.5 produced the highest wound closure among the tested membrane samples, whereas those containing 1% and 5% extract showed moderate effects. The untreated control displayed only minimal wound closure, confirming that the observed migratory effects were attributable to the extracts and their incorporation into the electrospun nanofibers.

### 2.9. Effect of EW7.5 Extracts on Wound Closure

**Figure 7:**
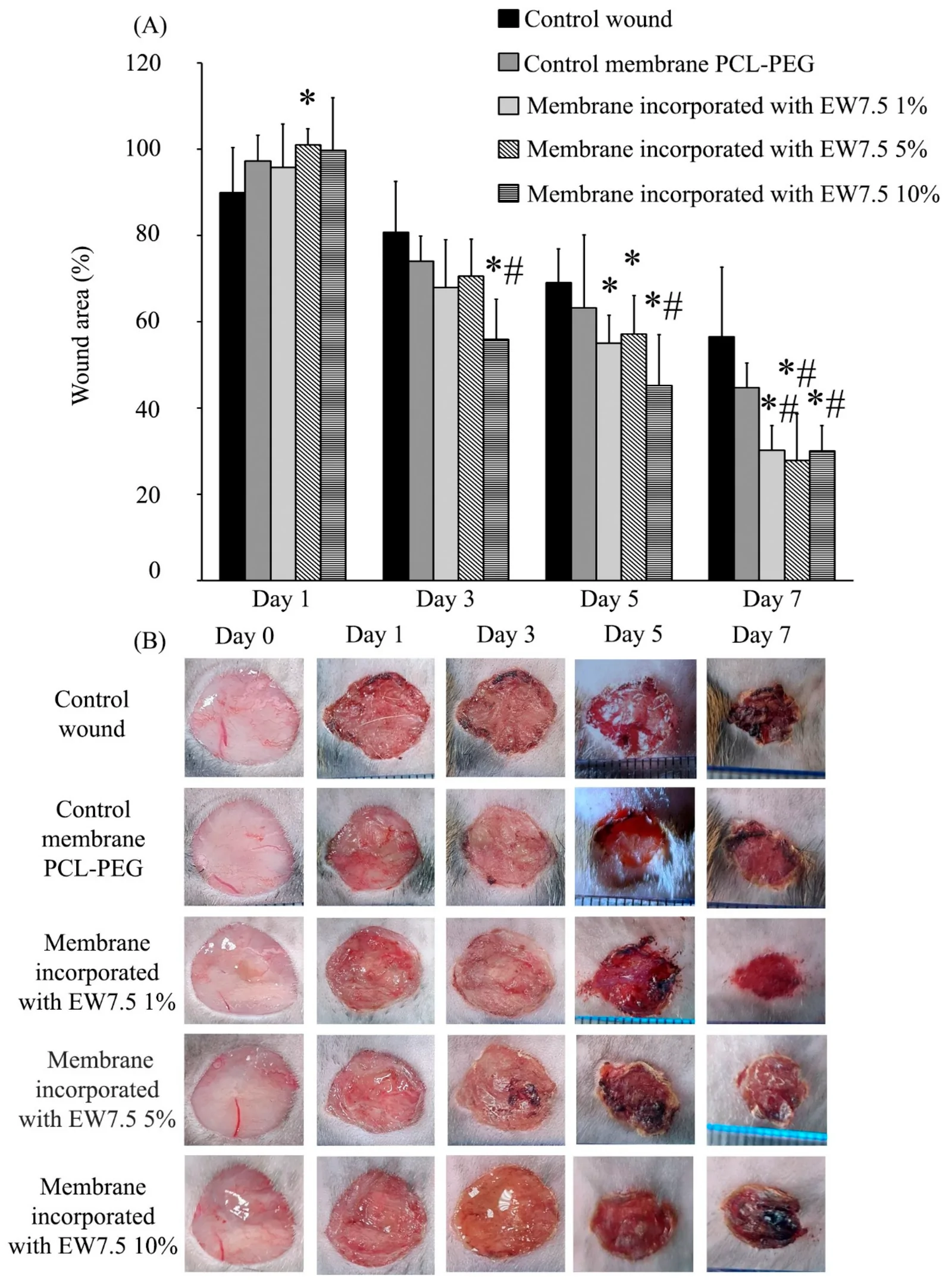
Dynamics of wound closure. (**A**) Wound closure is expressed as a percentage of the initial wound areas. (**B**) Representative photographs of wounds. The results were obtained from two independent experiments with five animals per group per experiment (a total of ten animals per group), and presented as mean ± standard deviation. Data were compared by the analysis of variance Kruskal–Wallis test followed by multiple comparisons of mean ranks (STATISTICA 7.0, StatSoft Inc, Tulsa, OK, USA). Statistically significant at * *p* < 0.05 compared to control wound group and # *p* < 0.05 compared to control membrane PCL-PEG group.

The aim of the in vivo part of the study was to examine if EW7.5 extracts incorporated in membranes can accelerate wound healing in a rat model of full-thickness wound. The latest study [44] showed that PCL-PEG electrospun nanofibers containing egg-yolk oil accelerate healing of full-thickness burns. While high efficiency of electrospun nanofibers has been shown in improving the wound-healing process [45], this efficiency is further increased in combination with plant extracts [46,47] due to their anti-inflammatory properties, including vervains. It has been shown that topical administration of the CO_2_ extract of this plant can accelerate wound healing (24 h and 48 h post wounding) in rats [13]. Our data showed significant decrease in wound areas between wounds covered with control PCL-PEG membranes and wounds covered with membranes incorporated with EW7.5 extracts. Decrease in wound area was noted on the third and fifth day in membranes incorporated with the EW7.5 extract 10% group compared to the control membrane PCL-PEG group, and in all examined concentrations of EW7.5 extract on the seventh day (Figure 7). After that, the wound areas were equalized.

**Figure 8:**
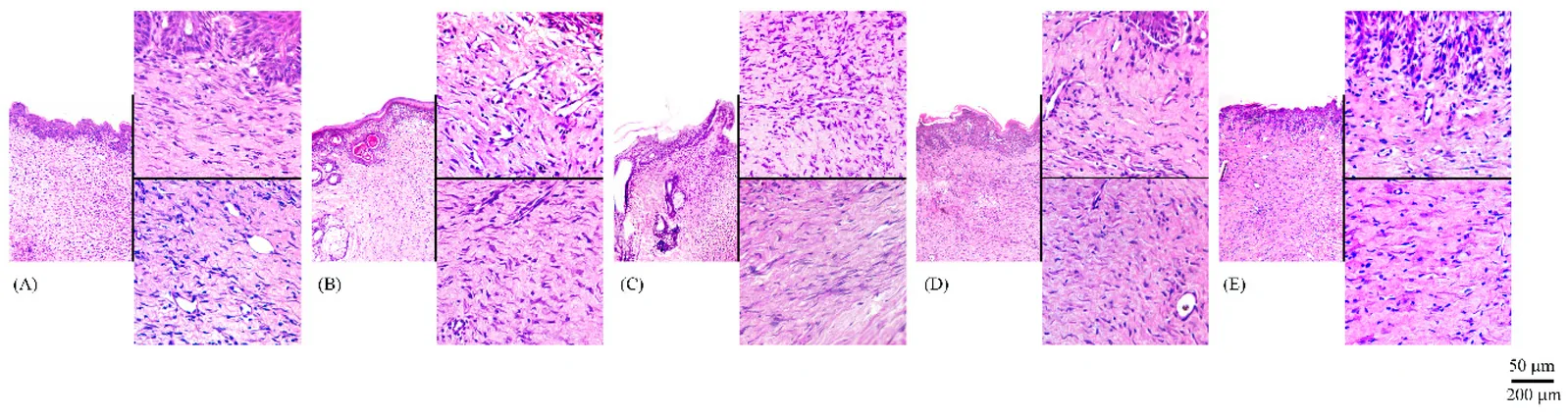
Light microscopy images of the wound tissue on day 7 after the injury. Representative photomicrographs of HE-stained full-thickness wounded skin in control (**A**), control membrane *PCL*-PEG group (**B**), membrane incorporated with EW7.5 extract 1% group (**C**), membrane incorporated with EW7.5 extract 5% group (**D**) and membrane incorporated with EW7.5 extract 10% group (**E**) animal groups. Scale bar = 200 μm for low-power field and 50 μm for high-power field.

Representative histological sections are shown in Figure 8. Seven days after injury, the untreated control and PCL–PEG membrane control groups showed similar histological features (Figure 8A,B), including moderately organized granulation tissue with fibroblasts and fine collagen fibers aligned parallel to the wound surface, thin-walled capillaries, and partial re-epithelialization. Semi-quantitative scoring confirmed no significant differences between these groups in inflammation (1.15 ± 0.50 vs. 1.40 ± 0.68), ECM deposition (2.00 ± 0.00 vs. 1.75 ± 0.45), vascularization (2.50 ± 0.41 vs. 2.75 ± 0.40), or re-epithelialization (1.50 ± 0.50 vs. 1.50 ± 0.48). EW7.5 extract-loaded membranes (Figure 8C–E), particularly the 5% and 10% groups, showed qualitatively more advanced healing, characterized by more mature granulation tissue with reduced cellularity and increased ECM deposition, narrower well-defined capillaries, more extensive re-epithelialization, and occasional hair follicles (Figure 8D,E). Semi-quantitative histological scoring supported these qualitative observations only for the EW7.5 10% group, which showed significantly greater ECM deposition (2.33 ± 0.41 vs. 1.75 ± 0.45, *p* = 0.04) and lower vascularization (2.17 ± 0.33 vs. 2.75 ± 0.40, *p* = 0.04) than the PCL–PEG membrane control, whereas re-epithelialization showed a non-significant trend toward improvement (2.00 ± 0.00 vs. 1.50 ± 0.48, *p* = 0.09). No significant differences in inflammatory scores were detected among the groups.

Recent studies increasingly support the use of plant extract-loaded electrospun nanofibers as multifunctional wound-dressing platforms. For example, electrospun PLGA/PMMA/collagen nanofibers loaded with *Syzygium cumini* leaf extract demonstrated enhanced antibacterial activity together with accelerated wound healing in both in vitro and in vivo models [36]. Likewise, *Rhamnus prinoides* leaf extract incorporated into PCL–cellulose acetate nanofibers exhibited improved antibacterial activity, favorable cytocompatibility, enhanced hydrophilicity and water absorption, and promoted in vitro wound closure [48]. These findings collectively demonstrate that the incorporation of phytochemicals into electrospun polymeric matrices represents an effective strategy for combining antimicrobial activity with improved wound repair. Within this context, the present study extends the field by integrating the comparative solvent-based selection of a chemically characterized *Verbena officinalis* extract, antimicrobial evaluation against clinically relevant skin isolates, incorporation into PCL–PEG electrospun nanofibers, LC–MS-based metabolite release monitoring, and biological validation through both in vitro and in vivo wound-healing models.

The superior biological performance of the EW7.5 extract is likely associated with the combined contribution of several major phytochemical classes identified by LC–MS analysis rather than with a single constituent. Iridoid glycosides, represented by verbenalin and hastatoside, have been associated with anti-inflammatory and tissue-protective activities, whereas phenylpropanoid glycosides such as verbascoside are well-known for their antioxidant, antimicrobial, and wound-healing properties. Likewise, luteolin derivatives^44^ and other flavonoids may contribute to the observed antimicrobial activity through membrane disruption, the inhibition of microbial enzymes, and the attenuation of oxidative stress, while phenolic acids provide additional antioxidant and anti-inflammatory effects. Therefore, the pronounced antimicrobial activity and enhanced wound-healing response observed for EW7.5 most likely reflect the complementary and potentially synergistic interactions among these phytochemical classes, rather than the action of a single dominant metabolite.

Despite the encouraging biological performance observed in the present study, several limitations should be acknowledged. The physicochemical characterization of the electrospun membranes was focused on parameters supporting proof-of-concept biological evaluation and did not include quantitative loading efficiency, comprehensive thermal or structural characterization, or quantitative release kinetics. Further development of the system will therefore require extended material characterization, quantitative release and stability studies, mechanistic evaluation, and validation in infected or chronic wound models. These steps, together with standardized extract production and reproducible membrane manufacturing, will be essential for further preclinical development and eventual clinical translation.

## 3. Material and Methods

### 3.1. Plant Collection

Samples of the plant material were purchased from the Institute for Medicinal Plants Research “Dr Josif Pancic”, Belgrade, Serbia. The aerial parts of *Verbena officinalis* (Verbenaceae) (*Verbenae herba*, product code: 602371, tea from the aerial parts of *Verbena officinalis* L., 100 g) was utilized and ground into a fine powder using a Waring 8010ES laboratory blender (Waring Commercial, Torrington, CT, USA).

### 3.2. Extractions

A 2 g sample was extracted overnight at 4 °C using 60 mL of each solvent listed in Table 6. On the following day, the samples were sonicated for 15 min and filtered through Whatman No. 4 paper. The plant residue was then re-extracted with an additional 60 mL of solvent at 4 °C over a period of 48 h, and this procedure was repeated. The extracts were subsequently evaporated to dryness at 40 °C using a rotary vacuum evaporator (Büchi R-210; Büchi Labortechnik AG, Flawil, Switzerland). The extraction procedure was performed independently in triplicate under identical conditions. The resulting extracts from the three batches were pooled to obtain a homogeneous sample, which was subsequently used for all chemical characterization, biological assays, and nanofiber fabrication, thereby minimizing batch-to-batch variability. The dried extracts were stored in airtight containers at −20 °C and protected from light and moisture, until further chemical and biological analyses. Dried extracts were dissolved in 99.5% methanol for chemical characterization and in 5% DMSO to prepare stock solutions for biological assays, which were subsequently diluted in the appropriate culture medium to the desired test concentrations.

### 3.3. UHPLC(−)HESI–QqQ-MS/MS Targeted Metabolomics Analysis

The extract’s metabolic profile was ascertained using the Liquid Chromatography–High-Resolution Tandem Mass Spectrometry (LC-HRMS/MS, Thermo Scientific™ Vanquish™ Core HPLC system) connected to the Orbitrap Exploris 120 mass spectrometer, San Jose, CA, USA. The Hypersil GOLDTM C18 analytical column (50 × 2.1 mm, 1.9 μm particle size) was part of the liquid chromatography system. The flow rate remained steady at 300 μL/min, and the injection volume was 5 μL. Ultrapure water + 0.1% formic acid (A) and acetonitrile (MS grade) + 0.1% formic acid (B) were used to elute the compounds of interest: 5% B for the first minute, 5–95% B for 1–10 min, 95% B for 10–12 min, and 5% B for 15 min.

An ESI source running in negative ionization mode was installed in the Orbitrap Exploris 120 mass spectrometer. While data-dependent MS2 tests were carried out at an Orbitrap resolution of 15,000 FWHM with a normalized collision energy of CID set to 35%, full scan MS was monitored from 100 to 1500 *m*/*z* with an Orbitrap resolution of 60,000 FWHM. The intensity threshold was set to 1 × 10^5^, and the dynamic exclusion duration was set to 10s with exclusion from a particular scan after two occurrences. Based on their chromatographic behavior, MS and MS2 comparisons with standard compounds, when available, and literature data that offered a preliminary identification, the phenolic compounds were identified. Data acquisition was carried out with Xcalibur^®^ data system (Thermo Finnigan, San Jose, CA, USA).

### 3.4. Antimicrobial Assays

#### 3.4.1. Isolation and Identification of Pathogenic Bacteria and Fungi from Infected Patients

Samples containing bacteria, yeasts, and fungi were collected at the Medical Military Academy (MMA), Institute of Microbiology and Immunology in Belgrade, Serbia, from patients suffering from skin and/or corneal infections over a one-year period, from October 2021 to October 2022. The strains were chosen based on their antibiotic sensitivity to commercial antibiotics/antimycotics as shown by standardized antibiogram protocol used in clinical practice [49]. A total of 16 human samples were obtained, and all patients provided informed consent for the use of their isolates in the experiments. The investigation was approved by the ethical committee of MMA (Approval No. 4/2021).

Microorganism identification was performed using a Vitek MS (bioMerieux SA, Marcy-l’Étoile, France) MALDI-TOF mass spectrometer.

#### 3.4.2. Antibacterial Activity

The strains used in the assay included skin isolates of bacteria: *Staphylococcus aureus* (ATCC 11632), *Staphylococcus lugdunensis* (B43), *Proteus vulgaris* (B44), and *Staphylococcus epidermidis* (B45). The antibacterial activity of *V. officinalis* extracts was evaluated using the microdilution method in 96-well microtiter plates. The inocula were prepared and standardized according to CLSI guidelines before each experiment. Minimum inhibitory concentrations (MIC) and minimum bactericidal concentrations (MBC) of the extracts against the bacterial species were determined as previously described [50,51]. Streptomycin served as positive control, while DMSO at the highest concentration used in the assays was used as a negative control. The assay was performed in triplicate.

#### 3.4.3. Antifungal Activity

The strains used in the assay included human isolates of dermatomycetes and yeasts collected as detailed in Section 3.4.1. These comprised two isolates of *Trichophyton mentagrophytes* (D448 and D465), two isolates of *Trichophyton rubrum* (D460 and D1026), one isolate of *Trichophyton violaceum* (D1182), two isolates of *Microsporum fulvum* (D89 and D351), two isolates of *Microsporum canis* (D277 and D371), as well as yeast isolates: *Candida albicans* (ATCC 10231), *Candida albicans* (Y177), *Candida krusei* (Y454), *Candida tropicalis* (Y149), and *Candida kefyr* (Y289). The antifungal activity was assessed using a modified serial dilution technique in 96-well microtiter plates.

Spores of dermatomycetes were harvested from 21-day-old cultures using a sterile 0.85% physiological solution containing 0.1% Tween (*v*/*v*). The spore suspension was adjusted to a concentration of 1.0 × 10^5^ spores per well using sterile physiological solution. The plates, containing the extracts, spores, and growth medium (Sabouraud Dextrose Broth (SDA)), were incubated at 37 °C for 72 h [52].

Minimum inhibitory concentrations (MICs) were defined as the lowest concentrations exhibiting no visible growth. Minimum fungicidal concentrations (MFCs) were determined by serially re-inoculating 10 µL from the wells without growth into 100 µL of SDA, followed by re-incubation for 72 h at 37 °C. The growth level or absence of growth was assessed by comparing treated wells to control wells, where dermatomycetes grew under the same conditions without the test extracts. The lowest concentration without visible growth was defined as the MFC. Minimum inhibitory concentrations (MIC) and minimum fungicidal concentrations (MFC) of the extracts against yeast species were determined as previously described [50,53]. Ketoconazole was utilized as a positive control, while DMSO at the highest concentration used in the assays was used as a negative control. The assay was performed in triplicate.

#### 3.4.4. The Activity of Extracts Against Biofilm Formation in *S. lugdunensis*

The impact of the extracts on *S. lugdunensis* (B43) biofilm formation was evaluated as previously described in the antimicrobial assay, with the exception that 96-well microtiter plates with an adhesive bottom (Sarstedt AG & Co. KG, Nümbrecht, Germany) were used. The concentrations of the extracts employed were the minimum inhibitory concentration (MIC), MIC/2, and MIC/4.

After 24 h of incubation, each well was washed twice with PBS, and the biofilms were fixed with methanol before air drying. The biofilm was then stained with 0.1% crystal violet (Bio-Merieux, Craponne, France) for 30 min. Following this, the wells were washed with water, air-dried, and the bound stain was dissolved in 100 μL of 96% ethanol (Zorka, Šabac, Serbia). The absorbance was measured at 620 nm using a Multiskan™ FC Microplate Photometer (Thermo Fisher Scientific, Waltham, MA, USA), and the percentage of inhibition of biofilm formation was calculated using the following formula:

[(A620 control − A620 sample)/A620 control] × 100.(1)

#### 3.4.5. The Activity of Extracts Against Formed Biofilm of *S. lugdunensis*

The assay was conducted as previously described [54]. *S. lugdunensis* was incubated in 96-well microtiter plates with an adhesive bottom (Sarstedt AG & Co. KG, Nümbrecht, Germany) in tryptic soy broth supplemented with 2% glucose at 37 °C for 24 h to establish biofilms. After incubation, the wells were washed twice with sterile PBS, and the remaining biofilm was treated with 2 × MBC, MBC, and MIC of the extracts (EW7.5, EW5, and EW2.5) for an additional 24 h at 37 °C. The procedure was subsequently repeated as outlined in Section 3.4.4.

### 3.5. Fabrication of Extract-Loaded Nanofiber Membranes

Extract-loaded nanofiber membranes were prepared through electrospinning. Based on preliminary studies, a polycaprolactone/polyethylene glycol (PCL/PEG) blend with a 75–25 ratio was dissolved in 2,2,2-trifluoroethanol at a concentration of 12.5% (*w*/*v*). To prepare PCL-PEG-extract solutions, three extract concentrations were tested: 1%, 5% and 10%. The extract was first dissolved in the solvent using an ultrasound bath until complete dissolution, after which PCL and PEG were added. The mixture was then stirred overnight on a magnetic stirrer until a homogeneous solution was obtained. Once prepared, the polymer solution was loaded into the syringe. The electrospinning process was performed at a voltage of 20 kV, with a needle-to-collector distance of 13 cm. The flow rate was set at 4 mL/h for solutions without the extract and 2 mL/h for extract-loaded solutions. Finally, the nanofibers were collected on aluminum foil wrapped around the drum collector. After the process, the fibers were carefully removed and dried in a vacuum desiccator for 24 h.

### 3.6. Characterization of Electrospun Nanofibers

The morphological characterization of the extract-loaded nanofibers was performed using scanning electron microscopy (SEM) with a Hitachi S-4800 (Hitachi High-Technologies Corporation, Tokyo, Japan), following a standard observation protocol. The chemical composition and functional groups present in the composite nanofibers were analyzed using Fourier Transform Infrared Spectroscopy (FTIR) with an NEXUS instrument, covering a spectral range of 4000–600 cm^−1^. Spectra were recorded at a resolution of 2 cm^−1^, averaging 64 scans for enhanced accuracy. The mechanical properties of the PCL/PEG blend, both with and without the extract, were evaluated using a Zwick/Roell Proline Z005 universal testing machine (ZwickRoell GmbH & Co. KG, Ulm, Germany) equipped with a 5 kN load cell. Tensile strength measurements were conducted with a preload of 0.03 N, followed by a pulling rate of 100 mm/min. Rectangular samples (40 × 5 mm) were carefully cut using a scalpel, and their thickness was measured to ensure consistency in mechanical testing.

### 3.7. In Vitro Release Study from Extract-Loaded Membranes (Lc–Ms Monitoring)

Release experiments were performed under sink-like conditions using an aqueous release medium (phosphate-buffered saline, PBS; pH 7.4) pre-equilibrated to 37 °C. Each membrane specimen (1%, 5%, or 10%) was placed in a sealed vial containing a fixed volume of release medium (kept constant for all samples), and incubated under agitation (e.g., orbital shaking) to minimize boundary-layer effects. At predetermined time points (15, 30, 60, 120, and 240 min, and 24 h), aliquots of the release medium were withdrawn for chemical analysis. The removed volume was immediately replaced with an equal volume of fresh, pre-warmed medium to maintain constant volume and concentration gradient (infinite dose conditions). Samples were clarified by filtration (0.22 µm PTFE) and stored at −20 °C until LC–MS analysis. Release aliquots were analyzed by LC–MS using the same chromatographic separation and MS settings as those applied for extract profiling. The 11 target compounds were monitored based on their retention times and accurate mass/characteristic ions. Peak areas were integrated using consistent parameters across all samples. The release was assessed semi-quantitatively and expressed as LC–MS peak area (arbitrary units).

### 3.8. Antibacterial Activity of Membranes Incorporated with Extracts

The antibacterial activity of the membranes was evaluated using the disk diffusion assay. Bacterial and fungal inoculums were prepared in the same manner as overnight bacterial/yeast cultures. A volume of 300 μL of the inoculum was evenly spread on Mueller–Hinton agar plates to create a uniform bacterial lawn. Thin film squares (10.0 × 10.0 mm) were then placed at the center of each Petri dish. The plates were incubated at 37 °C for 24 h. The antibacterial activity was assessed by measuring the zone of inhibition (mm). The net zone of inhibition was determined by subtracting the side length of the film square (10.0 mm) from the total inhibition zone observed around the test disk. Streptomycin served as the positive control, while blank films were used as the negative control.

### 3.9. Investigation of Extracts and Membranes Cytotoxicity

The cytotoxicity of the extracts (E1, EW7.5, EW5, EW2.5, and W1) and EW7.5-loaded electrospun membranes was evaluated using a crystal violet assay with slight modifications [29,55]. Human keratinocyte cells (HaCaT) were used as the in vitro model. HaCaT cells were seeded in 96-well plates at a density of 4 × 10^3^ cells per well and incubated for 48 h at 37 °C in a humidified atmosphere with 5% CO_2_ to allow cell adhesion. To assess the cytotoxicity of the membranes, leachate media were prepared by immersing membrane samples (20 × 30 mm; approximately 20 mg) in 4 mL of Dulbecco’s Modified Eagle Medium (DMEM) and incubating for 24 h at 37 °C. To assess the cytotoxicity of the extracts, E1, EW7.5, EW5, EW2.5, and W1 were dissolved and diluted in culture medium to the selected test concentrations (8 mg/mL). After cell adhesion, the culture medium was removed and replaced with 100 µL per well of either (i) membrane leachate medium or (ii) culture medium containing the tested extracts (E1, EW7.5, EW5, EW2.5, and W1). Untreated cells cultured in DMEM served as the negative control. Each condition was tested in triplicate wells and incubated for 24 h and 48 h.

Following treatment, the cells were washed twice with phosphate-buffered saline (PBS), and 100 µL of 0.4% crystal violet staining solution was added to each well and incubated at room temperature for 20 min. Excess dye was removed by washing under running tap water, and the plates were air-dried for 24 h. The bound dye was solubilized with 100 µL of methanol per well, and absorbance was measured at 570 nm (OD_570_) using a microplate reader. Cell viability was expressed as the relative growth rate (%) after 24 h and 48 h compared to untreated control cells.

### 3.10. In Vitro Wound- Healing (Scratch) Assay for Extracts and Membranes

The in vitro wound-healing (scratch) assay was performed to evaluate the effect of the tested extracts (E1, EW7.5, EW5, EW2.5, and W1) and EW7.5-loaded electrospun membranes on HaCaT keratinocyte migration [29]. HaCaT cells were cultured in DMEM and maintained at 37 °C in a humidified atmosphere containing 5% CO_2_. Cells were seeded in six-well plates and grown to 90–100% confluence. A linear scratch was created in each monolayer using a sterile 200 µL pipette tip. Detached cells were removed by gentle washing with PBS. For sample testing, cells were treated with medium containing each extract (E1, EW7.5, EW5, EW2.5, or W1) at a final concentration of 200 µg/mL. For membrane testing, leachate media were prepared by incubating electrospun membranes (20 × 30 mm; ~20 mg) in 4 mL of DMEM for 24 h at 37 °C, after which the conditioned medium was applied to the scratched monolayers. Untreated cells maintained in DMEM served as the control. Images of the wound area were captured immediately after scratching (0 h) and after 24 h of incubation using an inverted microscope. Wound closure was quantified using ImageJ 1.45 software by measuring the remaining cell-free area. The percentage of wound closure was calculated relative to the initial wound area (0 h). All experiments were performed in triplicate, and results were expressed as mean ± SD.

### 3.11. Animals

Animal treatment and experimental procedures were carried out in compliance with Directive 2010/63/EU on the protection of animals used for experimental and other scientific purposes and were approved by the Veterinary Directorate, Ministry of Agriculture, Forestry and Water Management (No 000692803 2025). The study was also conducted in adherence to the ARRIVE guidelines. Male Dark Agouti (DA) rats, 10–12 weeks old were conventionally housed (12 h light/dark cycle, ambient temperature of 22 ± 2 °C and 60% relative humidity) at the animal facility of the Institute for Biological Research “Sinisa Stankovic”, Belgrade, Serbia). During the experiments, the animals were kept in separate cages. They had unlimited access to standard rodent pelleted food and water. Animals were divided into five groups: (i) control wound (untreated wounds), (ii) control membrane PCL-PEG, (iii) membrane incorporated with EW7.5 extract 1%, (iv) membrane incorporated with EW7.5 extract 5%, and (v) membrane incorporated with EW7.5 extract 10%.

### 3.12. Wounding and Treatment with EW7.5 Extracts Incorporated in Membranes

The back of anesthetized animals (Vetflurane, Virbac, Carros, France, minimal alveolar concentration of 3% isofluran) was shaved, disinfected with 70% alcohol, and full-thickness excisional wounds (1 cm in diameter) were created by outlining the wound margins with a biopsy punch and subsequently excising the skin with surgical scissors. Animals were randomly allocated to the experimental groups. For pain management ketoprofen (KELA N.V., Hoogstraten, Belgium), 3 mg per kg of body weight, was administered by subcutaneous injection, before and 24 h after wounding. Polycaprolactone–polyethylene glycol (PCL-PEG) electrospun nanofibers (membranes) (1.5 cm in diameter) ± incorporated EW7.5 (1%, 5% and 10%) extracts were placed directly onto the wound surface and changed every day for five consecutive days. No additional fixation, such as sutures or bandages, was required, as the absorption of wound exudate allowed the membranes to remain adherent to the wound surface. The monitoring of wound closure included daily wound delineation using a plastic transparent sheet placed over the wound. The wound area was calculated from digitalized transparent sheets using ImageJ 1.45 software and changes were expressed as a percentage of the initial wound areas. Wound tissue was collected for further analysis on seventh day post wounding after euthanasia with Zoletil 100 overdose (Virbac, Carros, France), in line with other studies [56,57].

### 3.13. Histology

On day 7 after wounding, wound tissue samples were fixed in 4% formaldehyde, routinely processed for light microscopy, sectioned at 5 μm, and stained with hematoxylin and eosin (H&E). Sections were examined and photographed using a Leica DMLB light microscope (Leica Microsystems, Wetzlar, Germany) equipped with a Leica DFC295 camera and LAS Core software (v4.1.0). Histopathological evaluation was performed by an investigator blinded to the experimental groups. Inflammation, extracellular matrix deposition (ECM), vascularization, and re-epithelialization were semi-quantitatively scored on a 0–3 scale according to [58], where 0 = normal skin, 1 = discrete changes, 2 = moderate changes, and 3 = severe inflammation or high extracellular matrix deposition, vascularization, or re-epithelialization.

### 3.14. Statistical Analysis

The experiments were performed in three repeats, and results are presented as means  ±  SD. The data was statistically analyzed using GraphPad PRISM 9.0.0. software. The differences between control and experimental groups were compared using one-way ANOVA. A *p*-values (* *p* < 0.05, ** *p* < 0.01, *** *p* < 0.001, **** *p* < 0.0001) were considered to be statistically significant. The results from animal testing were obtained from two independent experiments with five animals per group per experiment (a total of ten animals per group). The required sample size was determined by a power analysis using G*Power 3.1.9.7 software, setting the threshold for significance (α) at 0.05, and statistical power at 0.95, and effect size was calculated based on the results of a preliminary study conducted with four samples per group (effect size f = 8.688). Results are presented as mean ± standard deviation. No animals or experimental data were excluded from the analysis. Data were compared by the analysis of variance Kruskal–Wallis test (STATISTICA 7.0, StatSoft Inc., Tulsa, OK, USA) and *p*-values < 0.05 were considered significant.

## 4. Conclusions

This study provides a comprehensive evaluation of the biological activity of *Verbena officinalis* extracts and their application in a nanofiber-based delivery system for skin-related therapeutic purposes. Among the investigated extracts, the hydroalcoholic extract (EW7.5) demonstrated the most pronounced antimicrobial and antibiofilm activity against both clinical isolates and reference strains of skin pathogens, including *Staphylococcus aureus* and *Candida albicans*. Chemical profiling and release studies confirmed the presence and sustained release of multiple bioactive metabolites from the electrospun nanofibers. The present work provides proof-of-concept evidence supporting the biological applicability of EW7.5-loaded electrospun PCL–PEG nanofibers. Nevertheless, additional physicochemical characterization, including porosity, swelling behavior, wettability, degradation, loading efficiency, extract distribution, residual solvent analysis, and quantitative release kinetics, will be necessary to further optimize the system and support its preclinical development.

Importantly, both the free extracts and EW7.5-loaded PCL–PEG nanofibers showed no cytotoxic effects on HaCaT keratinocyte cells at concentrations up to 400 µg/mL, indicating good in vitro biocompatibility. The in vitro wound-healing assay further demonstrated that EW7.5 significantly enhanced keratinocyte migration and wound closure, effects that were preserved after incorporation into nanofibers.

The in vivo evaluation using a DA rat excisional wound model confirmed the therapeutic potential of the EW7.5-loaded membranes, as they significantly accelerated wound closure and improved the histological features of healing compared to control membranes and untreated wounds. These results validate the traditional use of *V. officinalis* for skin disorders and provide modern experimental evidence supporting its incorporation into advanced biomaterial-based delivery systems.

The combination of antimicrobial efficacy, wound-healing activity, sustained release behavior, and favorable biocompatibility highlights the potential of EW7.5-loaded electrospun nanofibers as a promising candidate for the development of novel topical treatments for infected or chronic wounds.

## Figures and Tables

**Table 1:** Identified compound names, retention times (Rt, min), molecular formulas, MS data (calculated and exact masses, as well as mean mass accuracy—Δ mDa), major MS^2^ fragment ions of the compounds present in the extracts of *V. officinalis*.

No	Name	rt	Formula, M–H	Exact Mass	*m*/*z*	ppm	MS^2^
***Benzoic acid derivatives***							
1	Gallic acid	0.65	C_7_H_5_O_5_	169.01425	169.01427	−0.11	125.02480(8), 169.01764(100)
2	Vannilic acid hexoside	0.67	C_14_H_17_O_9_	329.08781	329.08793	−0.37	108.0217(14), 123.04523(29), 152.01135(18), 167.03497(100), 329.08878(15)
3	Dihydroxybenzoic acid hexoside	0.82	C_13_H_15_O_9_	315.07216	315.07224	−0.26	108.02192(4), 109.02962(13), 152.01181(11), 153.01968(36), 153.05571(100), 315.07187(14)
4	Gallic acid pentoside	1.05	C_12_H_13_O_9_	301.05651	301.05651	−0.01	125.02441(41), 168.00638(91), 169.01411(12), 283.04620(16), 301.05637(100)
5	Dihydroxybenzoic acid pentoside	1.90	C_12_H_13_O_8_	285.06159	285.06165	−0.21	108.02173(57), 109.02958(10), 152.01155(100), 153.01945(64), 285.06232(40)
6	Hydroxybenzoic acid isomer 1	2.29	C_7_H_5_O_3_	137.02442	137.02450	−0.58	93.03462(2), 137.02449(100
7	Benzoic acid	4.72	C_7_H_5_O_2_	121.02950	121.02957	−0.56	121.02962(100)
8	Dihydroxybenzoic acid pentosyl-pentoside	4.96	C_17_H_21_O_12_	417.10385	417.10359	0.63	108.02164(9), 109.02963(16), 152.01157(100), 153.01947(4), 241.07185(14), 285.06171(4)
9	Vanniloylmalic acid	5.61	C_12_H_11_O_8_	283.04594	283.04593	0.04	115.00375(11), 123.04518(25), 152.01146(4), 167.03499(100)
10	Syringoylmalic acid	5.73	C_13_H_13_O_9_	313.05651	313.05659	−0.27	71.01387(22), 115.00374(13), 138.03229(3), 153.05580(44), 182.02217(6), 197.04564(100)
11	Ellagic acid	6.07	C_14_H_5_O_8_	300.99899	300.99905	−0.18	300.99899(100)
12	Benzoylmalic acid	6.31	C_11_H_9_O_6_	237.04046	237.04042	0.18	71.01385(22), 115.0037(10), 121.02954(100), 149.06108(8)
13	Hydroxybenzoic acid isomer 2	6.46	C_7_H_5_O_3_	137.02442	137.02452	−0.76	93.03461(100), 137.02452(67)
***Cinnamic acid derivatives***							
**14**	Caffeic acid hexoside	4.85	C_15_H_17_O_9_	341.08781	341.08723	1.70	135.04520(21), 161.02426(17), 179.03513(100), 221.04575(24), 281.06757(13)
**15**	Chlorogenic acid	5.04	C_16_H_17_O_9_	353.08781	353.08710	2.00	191.05617(100)
**16**	Caffeic acid	5.13	C_9_H_7_O_4_	179.03498	179.03494	0.23	107.05049(3), 109.06586(6), 135.04517(98), 179.03494(100)
**17**	*p*-Coumaric acid hexoside	5.54	C_15_H_17_O_8_	325.09289	325.09289	0.00	119.05022(22), 145.02951(100), 161.06111(13), 163.04008(37), 187.03995(29), 205.05142(9)
**18**	Ferulic acid hexoside	5.68	C_16_H_19_O_9_	355.10346	355.10284	1.73	134.03717(20), 149.06123(21), 175.04013(71), 193.05058(100), 235.06139(64), 295.08136(18)
**19**	*p*-Coumaric acid	5.79	C_9_H_7_O_3_	163.04007	163.04008	−0.06	119.05029(100), 163.04018(12)
**20**	Lithospermic acid	6.07	C_27_H_21_O_12_	537.10385	537.10337	0.90	135.04535(100), 179.03485(48), 197.04573(100), 229.01418(47), 295.06116(88), 339.05032(70)
**21**	Ferulic acid	6.21	C_10_H_9_O_4_	193.05063	193.05053	0.53	134.03732(100), 149.0607(14), 178.02711(36), 193.05069(13)
**22**	Feruloylmalic acid	6.24	C_14_H_13_O_8_	309.06159	309.06156	0.11	115.00382(11), 133.01414(4), 134.03741(45), 149.06094(16), 178.02763(6), 193.05069(100)
**23**	Dicaffeoylquinic acid	6.37	C_25_H_23_O_12_	515.11950	515.11929	0.40	135.04514(8), 161.02461(4), 173.04553(11), 179.03496(53), 191.05608(100), 353.08713(7)
**24**	Rosmarinic acid	6.57	C_18_H_15_O_8_	359.07724	359.07678	1.29	135.04510(6), 161.02441(100), 179.03500(20), 197.04555(35)
**25**	Ethyl caffeate	7.27	C_11_H_11_O_4_	207.06628	207.06632	−0.18	134.03734(19), 135.04518(16), 161.02443(16), 179.03500(29), 207.06621(100)
***Iridoid glycosides***							
**26**	Verbraside	5.02	C_16_H_23_O_10_	375.12967	375.12908	1.56	89.02440(100), 101.02440(39), 113.02444(26), 119.03503(36), 151.07651(8), 195.06624(28)
**27**	Forsythide	5.24	C_16_H_21_O_11_	389.10894	389.10855	0.98	59.01381(100), 69.03458(76), 89.02440(55), 121.06585(52), 165.05534(27), 183.06606(7)
**28**	Ipolamiide	5.31	C_17_H_25_O_11_	405.14024	405.14014	0.23	121.06593(9), 149.06087(28), 181.08705(100)
**29**	Hastatoside + HCOOH	5.43	C_18_H_25_O_13_	449.13007	449.12949	1.28	101.02448(43), 139.04007(59), 209.04521(19), 223.06105(30), 241.07195(100)
**30**	Verbenalin + HCOOH	5.59	C_18_H_25_O_12_	433.13515	433.13472	1.00	59.01370(10), 89.02451(16), 101.02445(100), 119.03505(5), 193.05032(12), 225.07707(25)
**31**	Brasoside + HCOOH	5.62	C_17_H_23_O_11_	403.12459	403.12417	1.03	101.02419(12), 113.02480(12), 119.03480(13), 125.02451(100), 195.06592(4)
**32**	Forsythide dimethyl ester	5.86	C_18_H_25_O_11_	417.14024	417.14054	−0.72	153.05598(24), 181.01437(28), 193.12318(29), 197.04570(100), 237.11345(47), 402.11722(30)
**33**	Deoxyloganic acid	6.05	C_16_H_23_O_9_	359.13476	359.13420	1.54	59.01386(23), 89.02445(25), 109.06591(22), 135.08165(57), 153.09218(100), 197.08205(71)
***Phenylethanoids***							
**34**	β-Hydroxyacteoside	5.77	C_29_H_35_O_16_	639.19306	639.19279	0.43	113.02452(14), 135.04538(5), 151.04031(18), 161.02455(100), 179.03506(47)
**35**	Hellicoside	5.82	C_29_H_35_O_17_	655.18797	655.18797	0.00	113.02446(13), 135.04521(20), 161.02451(33), 179.03497(100)
**36**	Forsythoside B	6.04	C_34_H_43_O_19_	755.24040	755.24097	−0.76	135.04514(10), 161.02448(100), 179.03525(8), 461.16647(20), 593.20929(14), 623.19696(8)
**37**	Acetyl-verbascoside	6.10	C_30_H_37_O_16_	653.20871	653.20854	0.25	134.03755(8), 151.04019(51), 161.02487(35), 175.04013(100), 179.03491(13), 193.05069(90)
**38**	Verbascoside (Asteoside)	6.16	C_29_H_35_O_15_	623.19814	623.19788	0.42	113.02444(10), 135.04523(8), 161.02444(100), 461.16632(14)
**39**	Dehydroacteoside	6.28	C_29_H_33_O_15_	621.18249	621.18266	−0.28	113.02448(12), 135.04524(8), 151.04016(18), 161.02448(100), 179.03503(61)
**40**	Eukovoside	6.62	C_31_H_37_O_16_	665.20871	665.20893	−0.33	113.02451(12), 134.03731(25), 149.06081(7), 161.02440(16), 175.04005(27), 193.05060(100)
**41**	Martynoside	6.82	C_31_H_39_O_15_	651.22944	651.22957	−0.19	113.02446(15), 160.0166o(16), 175.04002(100), 193.05064(15)
***Flavonoid C-glycosides***							
**42**	Apigenin 6,8-di-*C*-hexoside	5.57	C_27_H_29_O_15_	593.15119	593.15132	−0.21	353.06604(100), 383.07687(66), 413.08545(9), 473.10886(47), 503.11929(15), 593.15131(28)
**43**	Apigenin 6-*C*-hexoside-8-*C*-pentoside	5.76	C_26_H_27_O_14_	563.14063	563.14054	0.16	353.06567(100), 383.07666(72), 413.08813(10), 443.09793(49), 473.10764(38), 563.13898(44)
**44**	Chrysin 6,8-di-*C*-hexoside	5.94	C_27_H_29_O_14_	577.15628	577.15617	0.18	337.07181(100), 367.08163(60), 457.11377(53), 487.12488(21), 577.15619(34)
***Flavonoid O-glycosides***							
**45**	Luteolin 7-*O*-(2″-hexuronyl)-hexuronide	5.76	C_27_H_25_O_18_	637.10464	637.10485	−0.33	113.02454(31), 193.03523(20), 285.04053(100), 351.0563(24)
**46**	Quercetin 3-*O*-hexoside	5.89	C_21_H_19_O_12_	463.08820	463.08832	−0.27	300.02756(50), 301.03525(100), 463.08823(15)
**47**	Quercetin 3-*O*-hexuronide	5.90	C_21_H_17_O_13_	477.06746	477.06721	0.54	113.02455(3), 151.00354(2), 178.99864(2), 301.03534(100)
**48**	Quercetin 3-*O*-(6″-rhamnosyl)-hexoside	6.03	C_27_H_29_O_16_	609.14611	609.14661	−0.83	151.00400(3), 300.02753(100), 301.03506(44), 609.14575(15)
**49**	Luteolin 7-*O*-hexoside	6.18	C_21_H_19_O_11_	447.09329	447.09278	1.12	284.03268(27), 285.04053(100), 447.09311(24)
**50**	Luteolin 7-*O*-hexuronide	6.19	C_21_H_17_O_12_	461.07255	461.07223	0.69	113.02444(4), 285.04044(100)
**51**	Apigenin 7-*O*-hexoside	6.45	C_21_H_19_O_10_	431.09837	431.09814	0.54	101.02439(15), 113.02452(10), 115.00379(15), 268.03772(97), 269.04556(66), 431.09845(100)
**52**	Isorhamnetin 3-hexuronide	6.46	C_22_H_19_O_13_	491.08311	491.08313	−0.02	113.02453(7), 151.00371(19), 175.04068(4), 287.05615(8), 300.02762(100), 315.05106(48)
**53**	Eriodictyol 7-*O*-hexoside	6.46	C_21_H_21_O_11_	449.10894	449.10851	0.95	101.02429(5), 125.02398(5), 135.04518(18), 151.00368(100), 287.05539(60)
**54**	Apigenin 7-*O*-hexuronide	6.50	C_21_H_17_O_11_	445.07764	445.07726	0.84	113.02442(15), 269.04547(100)
**55**	Myricetin 3-*O*-(acetyl)-rhamnoside	6.54	C_23_H_21_O_13_	505.09877	505.09875	0.03	271.02524(3), 316.02234(100), 317.02975(13)
**56**	Hispidulin 7-*O*-hexuronide	6.56	C_22_H_19_O_12_	475.08820	475.08817	0.07	113.02449(23), 284.03278(31), 299.05618(100)
**57**	Quercetin 3-*O*-(coumaroyl)-hexoside	6.58	C_30_H_25_O_14_	609.12498	609.12502	−0.06	300.02740(8), 301.03534(100)
**58**	Quercetin 3-*O*-(acetyl)-hexuronide	6.67	C_23_H_19_O_14_	519.07803	519.07775	0.54	113.02444(6), 151.00372(9), 178.99883(9), 300.02744(28), 301.03543(100)
**59**	Quercetin 3-*O*-(acetyl)-rhamnoside	6.93	C_23_H_21_O_12_	489.10385	489.10404	−0.39	300.02753(100), 301.03522(37)
**60**	Luteolin 3′-*O*-(3″-acetyl)-hexuronide	6.94	C_23_H_19_O_13_	503.08311	503.08307	0.08	285.04065(43), 503.08307(100)
***Flavonoid aglycones***							
**61**	Apigenin	6.49	C_15_H_9_O_5_	269.04555	269.04582	−1.00	269.04581(100)
**62**	Eriodictyol	7.01	C_15_H_11_O_6_	287.05611	287.05630	−0.65	107.01386(8), 125.02434(3), 135.04523(75), 151.00374(100)
**63**	Luteolin	7.08	C_15_H_9_O_6_	285.04046	285.04058	−0.40	151.00388(2), 285.04034(100)
**64**	Quercetin	7.09	C_15_H_9_O_7_	301.03538	301.03553	−0.51	121.02956(13), 151.00372(100), 178.99895(53), 257.04300(5), 273.04111(8), 301.03558(78)
**65**	Isorhamnetin	7.10	C_16_H_11_O_7_	315.05103	315.05116	−0.42	300.02750(100), 301.03033(3), 315.05099(10)
**66**	Naringenin	7.12	C_15_H_11_O_5_	271.06120	271.06132	−0.47	271.06116(100)
**67**	Syringetin	7.28	C_17_H_13_O_8_	345.06159	345.06156	0.09	287.01993(6), 315.01480(74), 330.03827(100)
**68**	Hispidulin	7.54	C_16_H_11_O_6_	299.05611	299.05620	−0.28	284.03247(100), 299.05591(30)
**69**	Tricin	7.60	C_17_H_13_O_7_	329.06668	329.06686	−0.55	299.01984(76), 314.04333(100), 329.06665(30)
**70**	Formononetin	7.92	C_16_H_11_O_4_	267.06628	267.06642	−0.51	252.04268(100), 267.06601(78)
**71**	Pinocembrin	7.98	C_15_H_11_O_4_	255.06628	255.06621	0.28	209.05914(10), 211.07646(14), 237.05540(28), 255.06593(100)
**72**	Cirsimaritin	8.05	C_17_H_13_O_6_	313.07176	313.07179	−0.09	269.04584(8), 283.02478(100), 298.04828(92), 313.07208(31)
**73**	Chrysin	8.42	C_15_H_9_O_4_	253.05063	253.05059	0.18	253.05064(100)
**74**	Genkwanin	8.45	C_16_H_11_O_5_	283.06120	283.06123	−0.11	268.03766(100), 283.06134(97)
***Xanthones***							
**75**	Trihydroxy-methoxyxanthone	7.56	C_14_H_9_O_6_	273.04046	273.04055	−0.32	258.01697(100), 273.04099(15)
**76**	Dihydroxy-trimethoxyxanthone	8.02	C_16_H_13_O_7_	317.06668	317.06667	0.03	287.01978(61), 302.04321(100)
**77**	Dihydroxy-methoxyxanthone	8.42	C_14_H_9_O_5_	257.04555	257.04558	−0.13	242.02214(86), 257.04550(100)
**78**	Dihydroxy-dimethoxyxanthone	8.51	C_15_H_11_O_6_	287.05611	287.05615	−0.13	257.00912(10), 272.03250(100), 287.05630(8)
**79**	Trihydroxy-dimethoxyxanthone	8.64	C_15_H_11_O_7_	303.05103	303.05107	−0.13	273.00418(100), 288.02762(91), 303.05161(26)
**80**	Trihydroxy-trimethoxyxanthone	8.99	C_16_H_13_O_8_	333.06159	333.06165	0.18	303.01465(89), 318.03812(100)

**Table 2:** Antimicrobial activity of different *Verbena officinalis* extracts; results are in mg/mL.

Extract		*S. aureus*	*S. lugdunensis*	*S. epidermidis*	*P. vulgaris*	*C. albicans* ATCC	*C. albicans* Y177	*C. krusei*	*C. tropicalis*	*C. kefyr*
E1	MIC	0.25	0.5	0.25	0.25	1	0.06	1	1	2
	MBC/MFC	0.5	1	0.5	0.5	2	0.125	2	2	4
EW7.5	MIC	0.06	0.06	0.06	0.06	0.06	0.06	0.06	0.06	0.06
	MBC/MFC	0.125	0.125	0.125	0.125	0.125	0.125	0.125	0.125	0.125
EW5	MIC	0.25	0.25	0.25	0.5	0.5	0.25	0.25	0.25	0.25
	MBC/MFC	0.25	0.5	0.25	1	1	0.5	0.5	0.25	0.5
EW2.5	MIC	0.25	0.5	0.25	0.125	0.25	0.125	0.06	0.125	0.125
	MBC/MFC	0.5	1	0.5	0.25	0.5	0.5	0.125	0.25	0.25
W1	MIC	0.5	2	2	1	1	2	2	2	2
	MBC/MFC	1	4	2	2	2	4	4	4	4
Streptomycin	MIC	0.003	0.003	0.1	0.003	–	–	–	–	–
	MBC	0.006	0.006	0.2	0.006	–	–	–	–	–
Ketoconazole	MIC	–	–	–	–	0.015	0.015	0.015	0.015	0.015
	MFC	–	–	–	–	0.030	0.030	0.030	0.030	0.030

**Table 3:** Anti-dermatomycetic activity of the extracts; results are in mg/mL.

Extract		*M. fulvum* (D89)	*M. fulvum* (D351)	*T. violaceum* (D1182)	*T. mentagrophytes* (D448)	*T. mentagrophytes* (D465)	*M. canis* (D277)	*M. canis* (D371)	*T. rubrum* (D460)	*T. rubrum* (D1026)
E1	MIC	1	1	1	2	1	1	1	1	1
	MFC	2	2	2	4	2	2	2	2	2
EW7.5	MIC	0.125	0.125	0.125	0.5	0.25	0.125	0.06	0.06	0.06
	MFC	0.25	0.25	0.25	1	0.5	0.125	0.125	0.125	0.125
EW5	MIC	1	1	1	2	2	1	1	1	1
	MFC	2	2	2	4	4	2	2	2	2
EW2.5	MIC	1	1	1	1	1	1	1	0.5	0.5
	MFC	2	2	2	2	2	2	2	1	1
W1	MIC	2	2	2	2	2	2	1	2	2
	MFC	4	4	4	4	4	4	2	4	4
Ketoconazole	MIC	0.025	0.025	0.0125	0.0125	0.0125	0.25	0.25	0.125	0.125
	MFC	0.050	0.050	0.025	0.025	0.025	0.50	0.50	0.025	0.025

**Table 4:** Diameters of inhibition zones (mm) for different pathogenic microorganisms when membranes incorporated with different concentrations of EW7.5 were applied.

Microorganism	1% EW7.5	5% EW7.5	10% EW7.5	Blank PCL–PEG
*Staphylococcus aureus* ATCC 11632	16.36 ± 0.68 ***	19.17 ± 1.11 ****	23.55 ± 0.95 ****	11.84 ± 0.24
*Staphylococcus lugdunensis* B43	18.28 ± 0.80 *	21.29 ± 3.49 **	27.25 ± 1.05 ****	12.54 ± 0.92
*Staphylococcus epidermidis* B45	15.34 ± 1.04 ns	25.65 ± 1.45 ***	31.62 ± 4.22 ****	11.74 ± 0.52
*Proteus vulgaris* B44	14.98 ± 1.44 ns	26.43 ± 1.33 ****	31.20 ± 1.46 ****	13.53 ± 1.89
*Candida albicans* ATCC 10231	14.72 ± 1.86 ns	17.94 ± 1.32 **	22.26 ± 1.94 ***	11.71 ± 0.46
*Candida albicans* Y177	15.66 ± 2.66 ns	17.24 ± 2.24 ns	29.99 ± 4.99 ***	12.18 ± 0.66
*Candida krusei* Y454	18.71 ± 1.09 *	18.53 ± 0.95 *	27.18 ± 3.06 ***	13.91 ± 1.73
*Candida tropicalis* Y149	15.04 ± 0.26 ns	18.32 ± 1.08 **	29.76 ± 2.26 ****	12.72 ± 1.16
*Candida kefyr* Y289	14.97 ± 1.07 ns	19.61 ± 0.80 ***	31.83 ± 2.31 ****	11.66 ± 1.22

Statistical significance was evaluated separately for each microorganism using one-way ANOVA followed by Dunnett’s multiple-comparison test, with blank PCL–PEG membranes as the control. * *p* < 0.05; ** *p* < 0.01; *** *p* < 0.001; **** *p* < 0.0001; ns, not significant.

**Table 5:** In vitro wound closure (%) of HaCaT cells after 24 h treatment with extracts and EW7.5-loaded electrospun membranes.

**Extract**	**Wound Closure (%)**
E1	33.01 ± 0.87
EW7.5	42.34 ± 1.58
EW5	37.21 ± 0.91
EW2.5	22.26 ± 2.48
W1	23.54 ± 0.86
**Membranes**	**Wound Closure (%)**
EW7.5 extract 1%	15.23 ± 1.25
EW7.5 extract 5%	17.76 ± 2.34
EW7.5 extract 10%	25.87 ± 2.52
Blank PCL-PEG	8.91 ± 1.73
Control	7.73 ± 0.07

**Table 6:** Extraction solvents used in this study.

Sample Code	Extraction Solvent	Extract Yield (% *w*/*w*)
E1	Ethanol 100%	13.58
EW7.5	Ethanol: Water 75%: 25%	18.27
EW5	Ethanol: Water 50%: 50%	15.42
EW2.5	Ethanol: Water 25%: 75%	12.79
W1	Water 100%	17.34

## Data Availability

The original contributions presented in this study are included in the article/Supplementary Material. Further inquiries can be directed to the corresponding author.

## Supplementary Materials

The following supporting information can be downloaded at https://www.mdpi.com/article/10.3390/ph19081278/s1, Figure S1. Benzoic acid derivatives; Figure S2. Cinnamic acid derivatives; Figure S3. Iridoid glycosides; Figure S4. Phenylethanoids; Figure S5. Flavonoid C-glycosides; Figure S6. Flavonoid O-glycosides; Figure S7. Flavonoid aglycones; Figure S8. Xanthones; Table S1. Release profile of selected metabolites from EW7.5-loaded PCL–PEG nanofiber membranes under physiological conditions; Table S2: Cytotoxic activity of tested extracts (E1, EW7.5, EW5, EW2.5, and W1) using the crystal violet assay on HaCaT keratinocyte cells.
