# Supplementary material for: Electrospun Nanofibers Loaded with Verbena officinalis Extract as Multifunctional Bioactive Wound Dressings
Source: Pharmaceuticals (Basel). 2026 Aug 13;19(8):1278. doi: 10.3390/ph19081278 (PMC13516329; doi:10.3390/ph19081278)
Supplement: Supplementary file 1 [file pharmaceuticals-19-01278-s001.zip › supplementary files Verbena (1).pdf]

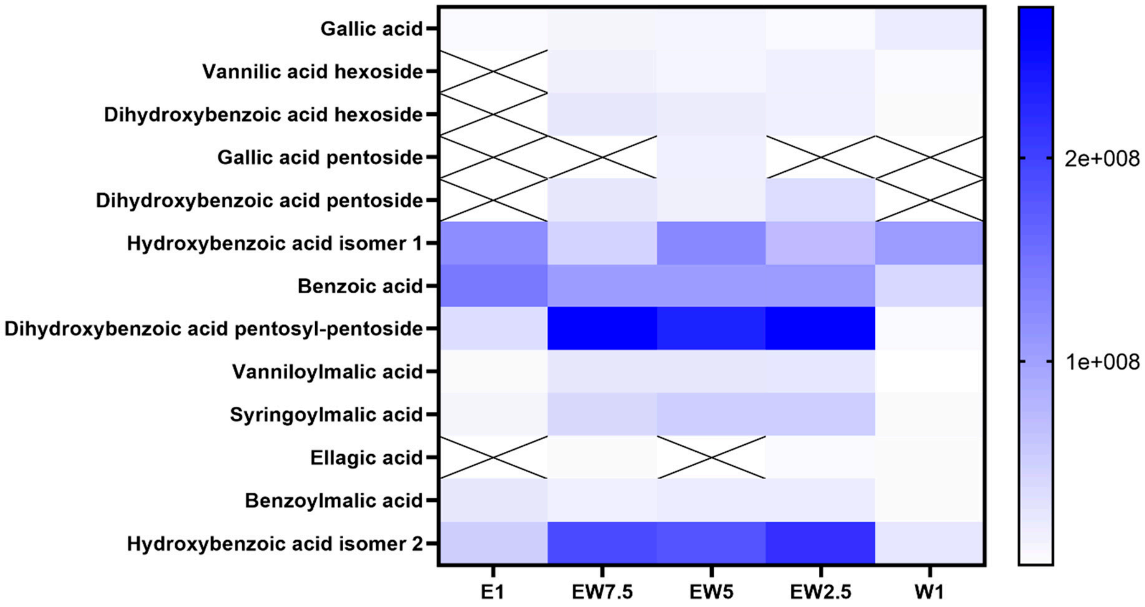

Figure S1. Benzoic acid derivatives.

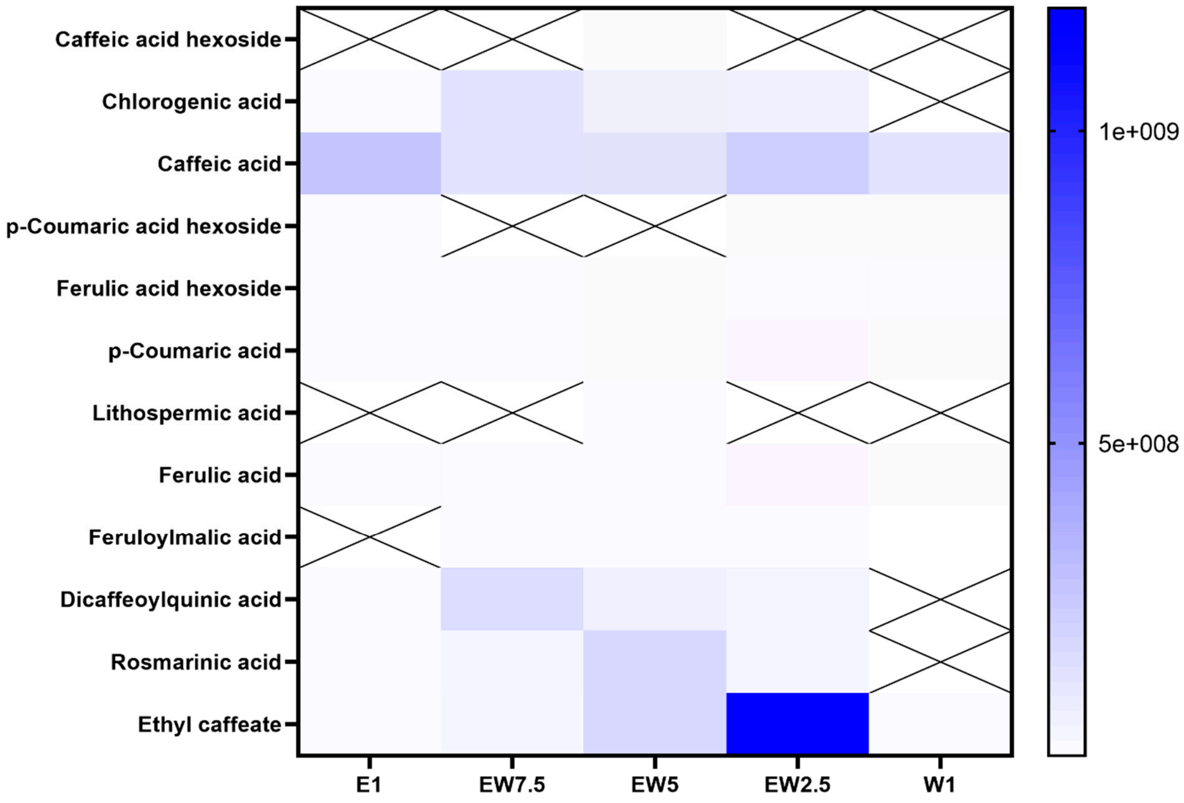

Figure S2. Cinnamic acid derivatives.

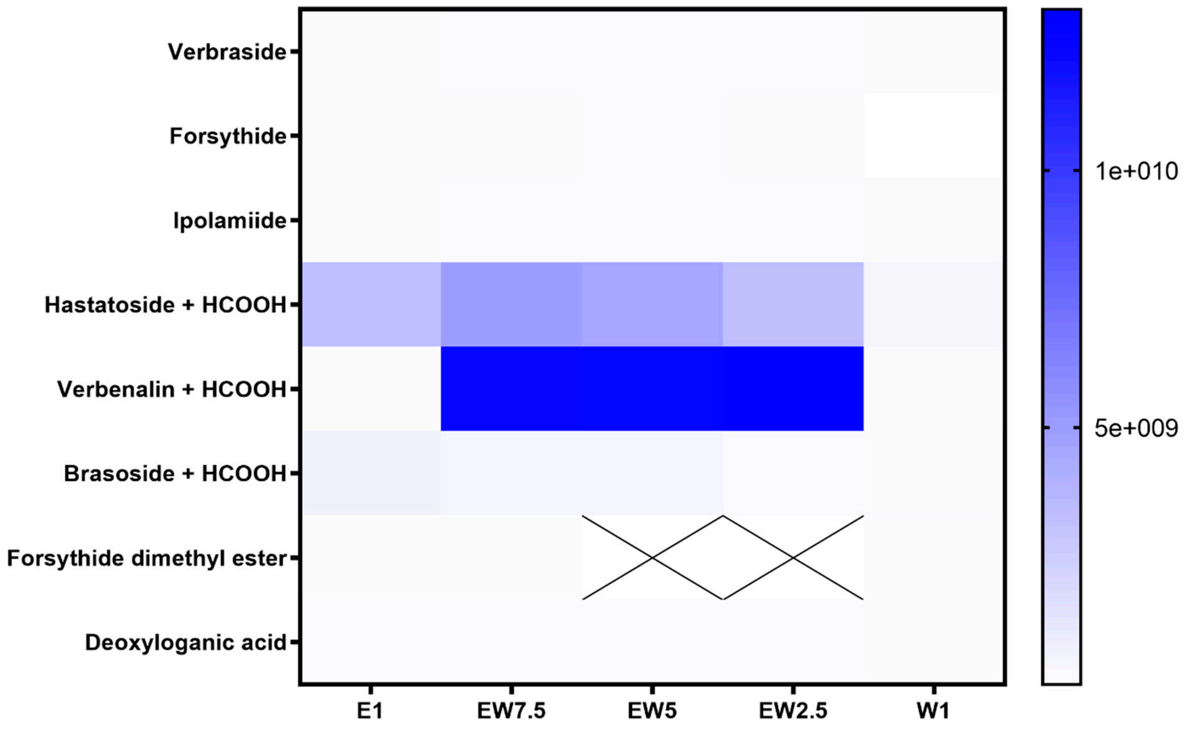

Figure S3. Iridoid glycosides.

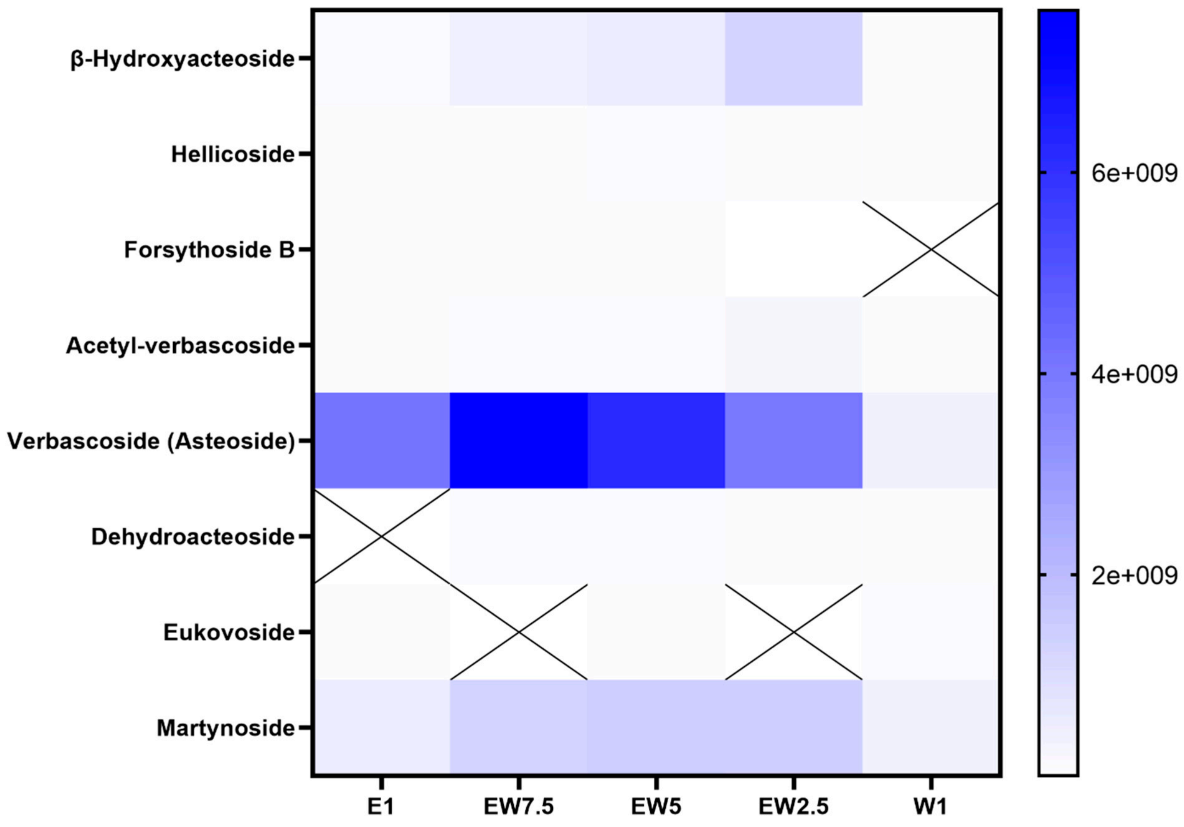

Figure S4. Phenylethanoids.

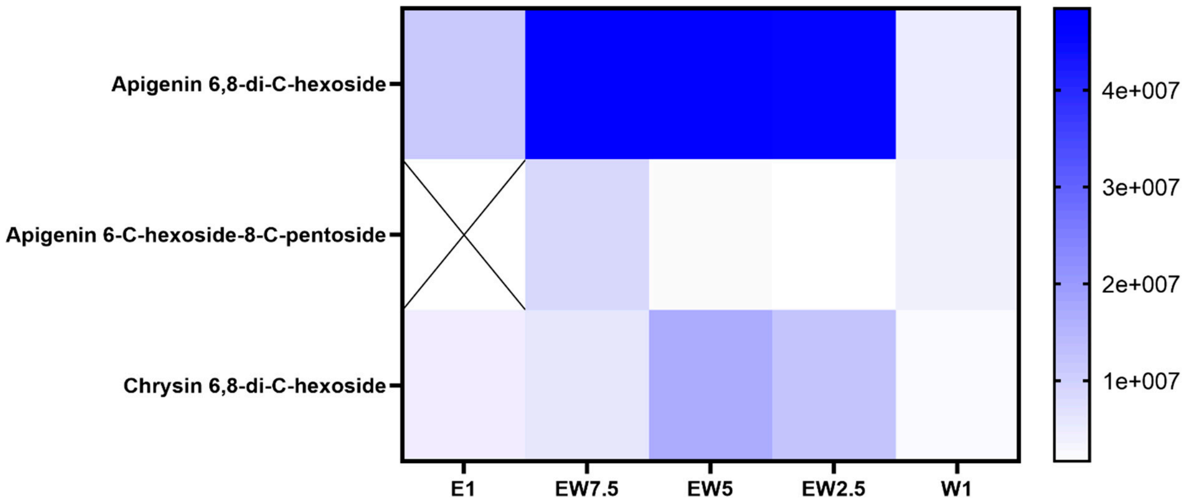

Figure S5. Flavonoid C-glycosides.

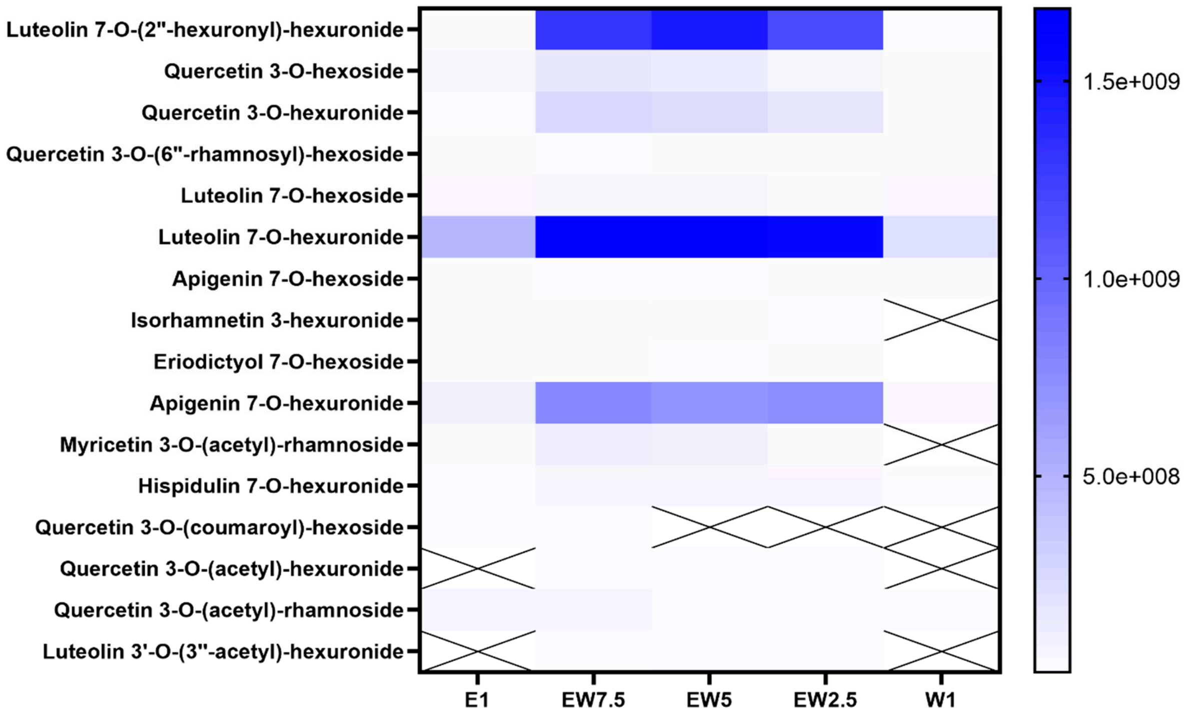

Figure S6. Flavonoid *O*-glycosides.

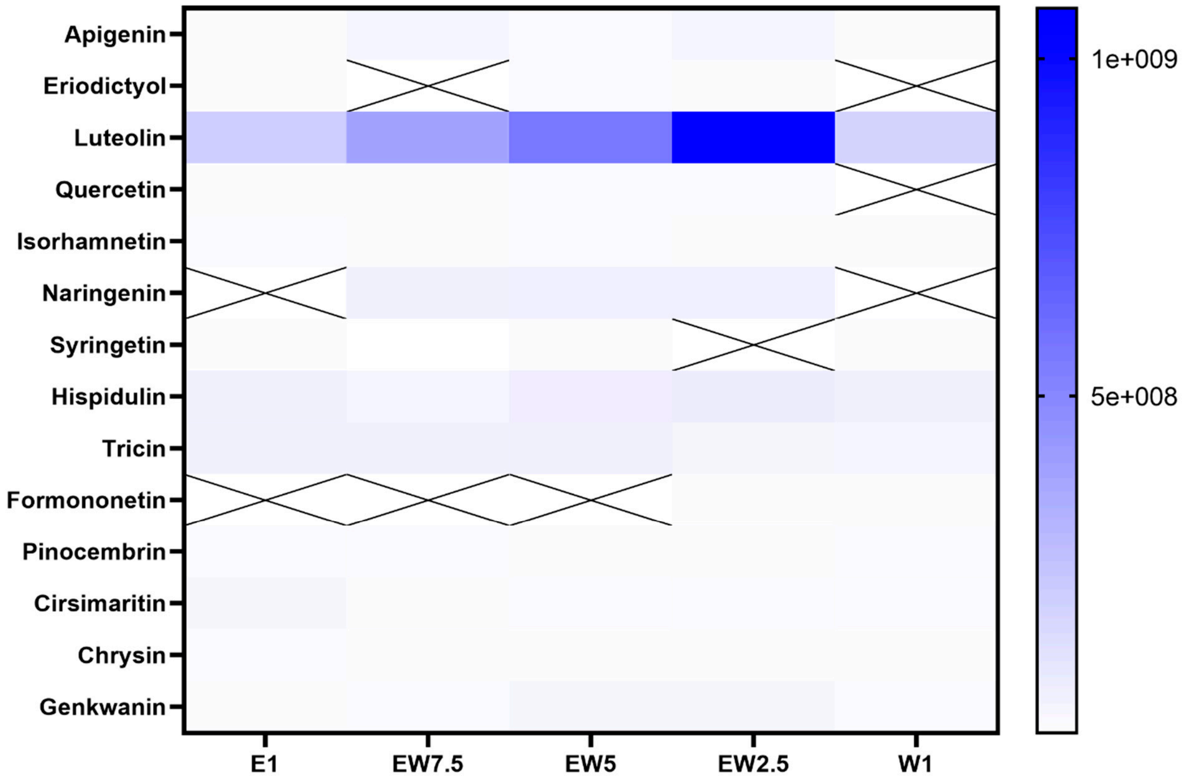

Figure S7. Flavonoid aglycones.

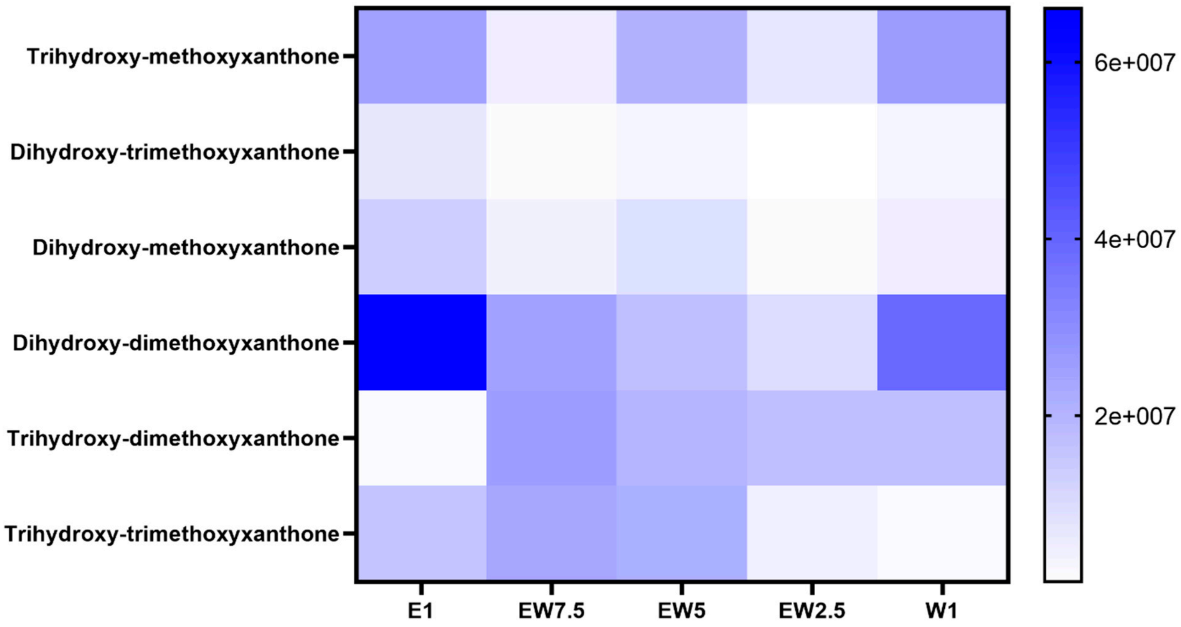

Figure S8. Xanthones.
