# Supplementary material for: Electrospun Nanofibers Loaded with Verbena officinalis Extract as Multifunctional Bioactive Wound Dressings
Source: Pharmaceuticals (Basel). 2026 Aug 13;19(8):1278. doi: 10.3390/ph19081278 (PMC13516329; doi:10.3390/ph19081278)
Supplement: Supplementary file 1 [file pharmaceuticals-19-01278-s001.zip › Supplementary table.pdf]

Table S2. Cytotoxic activity of tested extracts (E1, EW7.5, EW5, EW2.5, and W1) using the crystal violet assay on HaCaT keratinocyte cells.

Tested sample	IC_{50} (µg/mL)
**E1**	>400
**EW7.5**	>400
**EW5**	>400
**EW2.5**	>400
**W1**	>400
